# Integrated Zero By-Product Valorization of Orange Peel into Multifunctional Pectocellulosic-ZnO Nanocomposite Films for Sustainable Packaging

**DOI:** 10.3390/molecules31081297

**Published:** 2026-04-16

**Authors:** Safa Baraketi, Yosr Barchouchi, Cyrine Amara, Riadh Bez, Yassine M’Rabet, Ana Sanches Silva, Khaoula Khwaldia

**Affiliations:** 1 Laboratory of Natural Substances (LSN), National Institute of Research and Pysicochemical Analysis (INRAP), Sidi Thabet, Ariana 2020, Tunisia; safa.ca.25@gmail.com (S.B.); yosrbarchouchi73@gmail.com (Y.B.); cyrineamara16@gmail.com (C.A.); yassine.mrabet@gmail.com (Y.M.); 2 National Agronomic Institute of Tunisia, University of Carthage, 43 Charles Nicolle Avenue, Tunis 1082, Tunisia; 3 Laboratory of Materials Organization and Properties (LMOP), LR99ES17, Faculty of Sciences of Tunis, University of Tunis El Manar, Tunis 2092, Tunisia; riadh.bez@fst.utm.tn; 4 Faculty of Pharmacy, University of Coimbra, Polo III, Azinhaga de Stª Comba, 3000-548 Coimbra, Portugal; asanchessilva@ff.uc.pt; 5 Center for Study in Animal Science (CECA), ICETA, University of Porto, Apartado, 4050-453 Porto, Portugal; 6 Associate Laboratory for Animal and Veterinary Sciences (Al4AnimalS), 1300-477 Lisbon, Portugal

**Keywords:** orange peels, pectin, cellulose, ZnO nanocomposites, active packaging, circular bioeconomy

## Abstract

Agri-food waste valorization is critical for advancing sustainable packaging solutions. Citrus processing generates large amounts of peel residues that are often discarded, despite being rich in valuable biopolymers. This study presents a fully integrated, zero by-product valorization strategy for the fabrication and characterization of pectocellulosic nanocomposite films derived from orange peel (OP) biomass. Orange peel extract (OPE) was prepared and used for the biosynthesis of ZnO nanoparticles (NPs), while cellulose was obtained after the depectinization reinforced the pectin-rich supernatant used as the film-forming matrix (0–15% *w*/*w*). The optimized formulation containing 5% cellulose enhanced tensile strength by approximately 103% and reduced water vapor permeability by about 12.5% compared to the control, while maintaining structural homogeneity. Higher cellulose loading (≥10%) induced pore formation and compromised barrier and biological performance. Incorporation of ZnO NPs (1–5% *w*/*w*) into the optimized matrix further improved stiffness (YM = 163.9 MPa), improved UV-shielding capacity, antimicrobial activity (inhibition zones up to 16.0 mm), and antioxidant performance (98.2% ABTS inhibition). Biodegradation remained statistically unaffected by ZnO incorporation, with films retaining 29–35% degradation within 33 days. Overall, this work demonstrates the transformation of OP waste into multifunctional biodegradable active packaging materials, reinforcing circular bioeconomy principles.

## 1. Introduction

The global agri-food sector is characterized by immense productivity, but it is also responsible for generating vast quantities of by-products and waste. According to the Food and Agriculture Organization (FAO), nearly one-third of all food produced for human consumption, approximately 1.3 billion tons annually, is lost or wasted [1]. Fruits and vegetables, in particular, contribute about 45% of this total, with citrus fruits being among the most significant contributors due to their widespread cultivation and processing [2]. The industrial processing of citrus fruits, especially for juice production, results in substantial waste streams, predominantly peels, seeds, and pulp, which can constitute up to 50–60% of the fruit’s mass [3]. In 2023, global citrus production reached approximately 169.4 million tons, with oranges accounting for nearly half of this output [4]. Among this, up to 70% is processed into juice and concentrate, resulting in the production of over 32 million tons of citrus peel waste annually [5].

Despite the abundance of these by-products, recycling rates remain low. In most regions, only a minor portion is repurposed as animal feed, compost, or bioenergy, while the majority is relegated to landfills or open dumps. This underutilization not only represents a loss of valuable resources but also poses significant environmental challenges [6].

The disposal of citrus peel and other agri-food waste in landfills leads to several adverse environmental effects. The high organic content and low pH of citrus waste facilitate rapid microbial decomposition, resulting in the emission of greenhouse gases such as methane (CH_4_) and carbon dioxide (CO_2_) [6]. Methane emissions from landfills are particularly concerning, as methane is a potent greenhouse gas with a global warming potential 25 times greater than CO_2_ over a 100-year period [6]. In addition, leachate from decaying citrus waste can contaminate soil and groundwater, while the accumulation of organic matter promotes eutrophication, foul odors, and the proliferation of disease vectors [5].

Recent life cycle assessment (LCA) studies have quantified the environmental burdens associated with different waste management strategies. For example, the global warming potential (GWP) of food waste ranges from 347 to 2969 kg CO_2_ equivalent per ton, depending on the waste type and management system. Citrus peel waste, when landfilled, can generate up to 6.4 kg CO_2_ eq per kg of waste. These impacts underscore the urgent need for innovative valorization pathways that transform waste into valuable products while mitigating environmental harm [7].

Moreover, this disposal practice overlooks the fact that agri-food by-products are rich in valuable compounds, including bioactive molecules, fibers, and polysaccharides, which can be harnessed for sustainable applications. In particular, the development of biodegradable packaging materials from agri-food waste has emerged as a promising strategy to reduce reliance on petroleum-based plastics while simultaneously valorizing waste streams.

Orange peel (OP) is a highly valuable resource, containing a wide range of bioactive compounds such as flavonoids (hesperidin, naringin), polymethoxylated flavones (nobiletin, tangeretin), phenolic acids (ferulic, caffeic, p-coumaric), carotenoids, and essential oils (limonene, linalool) [8]. These compounds are known for their antioxidant, antimicrobial, and anti-inflammatory properties, making citrus waste an attractive raw material for functional packaging. In addition to these low-molecular-weight bioactive compounds, OP is rich in structural biopolymers. Among them, pectin and cellulose are the main polysaccharides used for biodegradable film development.

Pectin is a complex heteropolysaccharide composed mainly of galacturonic acid units. In citrus peel, pectin represents approximately 20–30% of the dry weight [9]. Traditionally used in the food industry as a gelling and stabilizing agent, pectin has gained attention in packaging research due to its ability to form transparent, flexible, and biodegradable films. Pectin films can also act as carriers of bioactive compounds, imparting antioxidant and antimicrobial properties to packaged foods [10]. However, pure pectin films often suffer from poor mechanical strength and high water sensitivity, necessitating reinforcement with other biopolymers or fillers [11]. Moreover, most reported pectin-based films rely on commercially purified pectin, which limits the sustainability and economic feasibility of large-scale waste valorization strategies.

Cellulose, being the most abundant biopolymer in citrus peels, is also present in OP at levels of up to 30% of the dry weight [12]. Composed of linear chains of β-(1→4)-linked glucose units, cellulose provides rigidity to plant cell walls and, when extracted, can be used to reinforce biopolymer films. Its high mechanical strength, barrier properties, and biodegradability make cellulose an ideal additive for packaging applications [13]. When incorporated into pectin films, cellulose can reduce water vapor permeability, improve tensile strength, and enhance structural stability, thereby overcoming the limitations of pure pectin films [14]. Despite these advantages, previous studies have typically combined pectin and cellulose from different sources or incorporated externally supplied fillers, rather than integrating both polymers from a single biomass stream within a unified valorization process [11,14].

Moreover, OP is increasingly recognized as a valuable resource in nanotechnology, as it is rich in bioactive compounds such as polyphenols and flavonoids that can be used directly as active additives or serve as precursors for the synthesis of inorganic nanoparticles. These natural extracts act as reducing and stabilizing agents, enabling the sustainable production of nanomaterials while simultaneously valorizing food waste. Among inorganic nanoparticles, zinc oxide (ZnO) has attracted broad interest across diverse domains, including electronics [15], photocatalysis [16], solar cells [17], and biomedical applications [18], due to its wide band gap, high exciton binding energy, and strong antimicrobial activity [19]. More recently, ZnO nanoparticles (ZnO NPs) have been explored as active fillers in biopolymer-based food packaging, where they enhance mechanical strength, barrier properties, UV shielding, and microbial resistance [20]. The synthesis method and particle size of ZnO NPs are crucial determinants of their performance. Conventional chemical routes such as sol–gel, precipitation, and hydrothermal methods allow precise control but often involve toxic reagents. In contrast, green synthesis using agri-food waste, including fruit peels, offers an eco-friendly alternative that produces biocompatible ZnO NPs with tunable properties. Parameters such as precursor concentration, pH, reaction temperature, and reaction time strongly influence particle size and crystallinity, with smaller NPs exhibiting higher surface reactivity, better dispersion in polymer matrices, and stronger antimicrobial and photocatalytic activity [21].

Recent studies have demonstrated significant progress in the development of pectin-based composites reinforced with cellulose and ZnO NPs for food packaging applications. The incorporation of cellulose, particularly in micro- or nanoscale forms, into pectin matrices has been shown to significantly improve mechanical strength, reduce water vapor permeability, and enhance film stability, thereby addressing the inherent limitations of pure pectin films [22]. However, these systems often rely on externally sourced cellulose, limiting their sustainability and integration potential. In parallel, ZnO NPs have been widely investigated as multifunctional additives in biopolymer-based packaging due to their strong antimicrobial activity, UV-shielding capacity, and ability to improve thermal and mechanical properties. For instance, pectin-based films reinforced with ZnO NPs have shown effective inhibition of foodborne pathogens while maintaining structural integrity, thus contributing to extended shelf life [14,23,24].

More recently, the combined incorporation of cellulose nanocrystals and ZnO NPs into pectin matrices has been shown to produce multifunctional materials with enhanced mechanical, barrier, and antimicrobial properties [14]. Nevertheless, these approaches generally involve multi-source systems and lack full integration within a single biomass valorization pathway.

Building on the complementary properties of pectin and cellulose, the present study proposes an integrated and original valorization strategy in which both polymers are extracted from the same agri-food waste matrix, OP, and subsequently recombined to form functional biodegradable films. Unlike most previous studies that rely on commercially purified polymers or external reinforcements, this work adopts an integrated zero by-product valorization approach, in which the pectin-rich supernatant is used as the film-forming matrix while the recovered cellulose is reincorporated as a reinforcing phase. Furthermore, to extend the functionality of the optimized pectocellulosic system, ZnO NPs were biosynthesized using orange peel extract (OPE) and incorporated as an active nanofiller, thereby creating a fully biomass-integrated nanocomposite platform. The main objective of this study is to develop and characterize pectocellulosic composite films derived entirely from OP waste and to evaluate the effect of cellulose content on their structural, optical, barrier, biological, and biodegradability properties. Subsequently, the influence of biosynthesized ZnO NPs on the mechanical performance, functional activity, and environmental degradability of the optimized formulation was investigated. Through this approach, the study aims to demonstrate the feasibility of producing fully bio-based, functional packaging materials while advancing circular bioeconomy-oriented strategies for agri-food waste valorization.

## 2. Results and Discussion

### 2.1. Characterization of Orange Peel Powder

The lignocellulosic characterization of OP powder shows that this biomass contains 31.42 ± 0.42% α-cellulose and 26.69 ± 1.06% pectin, corresponding to an overall pectocellulosic fraction of 58.11% on a dry weight basis. This composition was compared with values reported in the literature (Table 1), revealing a profile that is favorable for pectocellulosic bioplastic fabrication due to its relatively high pectin and cellulose contents [12,25]. The low hemicellulose content (3.80 ± 0.54%) compared to that reported by Ayala et al. [26] indicates reduced structural recalcitrance, which may facilitate polymer extraction and film formation. The ash (3.84 ± 0.13%), moisture (9.41 ± 0.27%), and soluble protein contents (1.7 ± 0.15%) are consistent with previously reported studies (Table 1). Furthermore, in the present work, extractives accounted for 32.60 ± 0.52% of the raw material, which is comparable to values reported in the literature. For instance, Ortiz-Sanchez et al. [12] reported an extractives content of approximately 30%. These differences can be attributed to several factors, including varietal differences and environmental and agronomic conditions (soil, climate, irrigation, and fertilization), as well as harvest timing and fruit maturity, all of which can significantly influence the relative proportions of extractable and structural polysaccharides.

**Figure 1:**
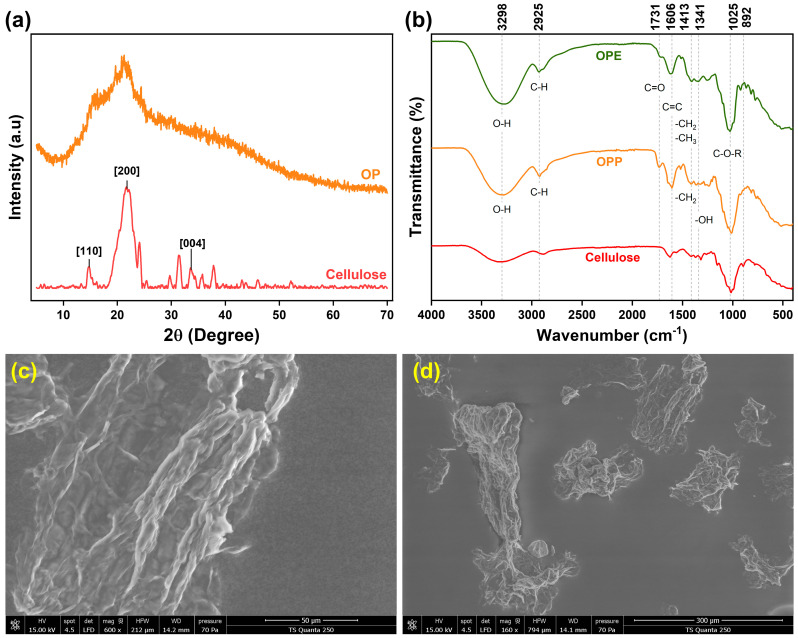
(**a**) XRD patterns of OP and extracted cellulose; (**b**) FTIR spectra of OP, cellulose, and OPE; and (**c**,**d**) SEM micrographs of cellulose extracted from OP.

The XRD pattern of OP revealed a broad hump centred around 2θ ≈ 20°, with no sharp and intense diffraction peaks (Figure 1a). This feature is characteristic of predominantly amorphous materials and indicates the absence of long-range crystalline order in the raw biomass matrix. The amorphous halo is typically associated with the presence of pectin, hemicellulose, and other non-crystalline organic constituents, which are known to exist largely in disordered states. Although cellulose is present in OP, its crystalline domains are likely masked by the dominant amorphous components and the complex lignocellulosic structure. Similar findings have been reported in citrus peel biomass [27], where broad diffraction halos in the range of 18–22° confirmed the largely amorphous nature of the phytochemical and polysaccharide matrix.

**Table 1:** Chemical composition of orange peel powder (wt%).

	This Study	Other Studies	References
α-Cellulose (%)	31.42 ± 0.42	30.17 ± 0.50	[12]
Hemicellulose (%)	3.80 ± 0.54	5.43 ± 0.05	[26]
Pectin (%)	26.69 ± 1.06	24.25 ± 0.24	[25]
Extractible (%)	32.60 ± 0.52	30.76 ± 1.07	[12]
Soluble protein (%)	1.70 ± 0.15	1.95	[28]

The data are shown as mean values ± s.d. Moisture content of the raw OP powder was 9.41 ± 0.27% (wet basis).

### 2.2. Characterization of OP-Derived Materials and ZnO NPs

#### 2.2.1. Orange Peel Extract (OPE)

The FTIR analysis of OPE showed characteristic absorption bands corresponding to the functional groups present in the raw material (Figure 1b). A prominent peak at 3276 cm^−1^, is indicative of O-H stretching vibrations from hydroxyl groups in alcohols and phenolic compounds. In addition, the distinct band at 2925 cm^−1^ is assigned to C–H stretching vibrations, while the absorption band at 1413 cm^−1^ is attributed to C–H bending vibrations. Both signals arise from –CH_2_- and –CH_3_ aliphatic chains. The presence of carbonyl groups (C=O) is associated with an absorption around 1730 cm^−1^, corresponding to ester or carboxylic acid groups, commonly found in pectin and other polysaccharides. Furthermore, the band at 1616 cm^−1^ is linked to C=C vibrations, reflecting flavonoids and other aliphatic and/or aromatic compounds. Finally, an intense band at 1025 cm^−1^ indicates the presence of C-O-H and/or C-O-C linkages, particularly alcohols and esters [29,30]. These results provide valuable insights into the molecular constituents of OPE.

The aqueous ethanolic OPE exhibited a total phenolic compound (TPC) of 148.39 ± 2.65 mg GAE/g extract, confirming its richness in polyphenols. Antioxidant activity was further assessed using DPPH radical scavenging (33.09 ± 3.55 mg TE/g extract), ABTS assay (97.77 ± 5.19 mg TE/g extract), and FRAP (61.78 ± 1.41 mg TE/g extract). These values highlight the strong reducing potential of OPE, supporting its role as a biogenic agent for ZnO NPs synthesis. Comparative studies show that the TPC value was higher than that reported by Haya et al. [31], who found 111.84 and 17.5 mg GAE/mL when extracted with water and 70% ethanol, respectively. On the other hand, Akhabue et al. [32] found a TPC of approximately 271 mg GAE/g for ethanolic extracts. Regarding antioxidant activity, the ABTS value (97.77 ± 5.19 mg TE/g) exceeded those reported by Zaghbib et al. [33] for citrus peel extracts, whereas the DPPH value obtained was slightly lower than those reported in some studies. These differences can be attributed to variations in solvent composition, cultivar, and extraction conditions. Overall, the relatively high TPC and strong ABTS/FRAP activities confirm that OPE possesses significant antioxidant capacity, validating its use as a reducing and stabilizing agent in green ZnO NP synthesis.

#### 2.2.2. OP-Derived Cellulose

The FTIR spectra of OP and extracted cellulose reported in Figure 1b show a broad absorption band at 3298 cm^−1^ attributed to hydroxyl O–H stretching vibrations. This peak reflects the extensive hydrogen bonding network within cellulose. The absorption bands around 2900 cm^−1^ and 1370 cm^−1^ correspond to C–H group stretching and deformation vibrations in glucose units [34]. The bands at 1731 cm^−1^ and 1520 cm^−1^ are assigned to aromatic C=C ring stretching and C–H deformation in lignin methylene, methyl and methoxyl groups. These peaks are absent in the spectrum of extracted cellulose, confirming that the majority of hemicellulose and lignin were effectively removed during alkaline and bleaching treatments of OP powder [35]. The absorption band at 1624 cm^−1^ is assigned to the adsorbed water. Remaining bands at 1417, 1315, 1155 and 1025 cm^−1^ correspond to the –CH_2_ scissoring, –OH bending, C–O antisymmetric and C–O–C pyranose ring skeletal vibrations of cellulose, respectively [34]. Moreover, the absorption at 892 cm^−1^ is characteristic of β-glycosidic linkages, confirming the presence of the cellulose polysaccharide backbone [36]. Notably, the FTIR spectrum of OP cellulose exhibits the same set of absorption bands as the commercial cellulose cited by Abderrahim et al. [36], confirming the structural similarity and validating the successful extraction of cellulose with preserved chemical integrity.

The XRD diffractogram of cellulose (Figure 1a) exhibited three characteristic reflections at 2θ = 14.7° (110), 21.8° (200), and 33.8° (004), which are typical of cellulose I crystalline structure. The (110) peak corresponds to the lateral packing of cellulose chains, reflecting hydrogen-bonded sheet arrangements. The (200) peak, the most intense reflection, represents the crystalline plane aligned with the chain axis and is the primary indicator of crystallinity [37]. The (004) peak at higher angles corresponds to the periodicity along the c-axis of the unit cell, providing information about long-range order in crystalline domains [38,39]. The cellulose sample exhibited a CI of 70.29%, indicating a high proportion of ordered crystalline regions. This value falls within the range reported for plant-derived cellulose (55–75%), confirming that the cellulose retains significant structural order [40]. A high CI is associated with improved mechanical strength, thermal stability, and reduced water uptake, making cellulose an effective reinforcement agent in biopolymer films.

The SEM micrograph of the cellulose (Figure 1c) revealed a distinct fibrous morphology characterized by ribbon-shaped, short fiber stands arranged in irregular fragments (Figure 1d) and an interconnected network structure. Alkaline pretreatment and bleaching process enhance fibrillation, exposing microfibrillar bundles and increasing surface roughness.

#### 2.2.3. ZnO Nanoparticles

**Figure 2:**
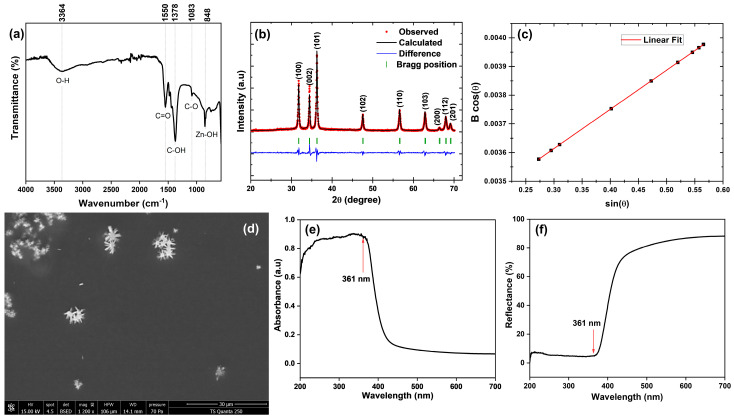
(**a**) FTIR spectrum of biosynthesized ZnO NPs using OPE, (**b**) Rietveld analysis of the X-ray diffraction pattern of the ZnO sample, (**c**) Williamson-Hall plot of ZnO sample, (**d**) SEM images of ZnO NPs, (**e**) solid state UV-Vis absorbance spectrum, and (**f**) diffuse reflectance spectrum of ZnO NPs.

The FTIR spectrum of ZnO NPs synthesized using OPE (Figure 2a) revealed distinct absorption bands that confirm the involvement of phytochemicals in nanoparticle formation and stabilization. A broad peak at 3364 cm^−1^ corresponds to O–H stretching vibrations, indicative of hydroxyl groups from polyphenols and alcohols present in the extract, which act as capping agents. The band at 1550 cm^−1^ is attributed to C=O stretching of carbonyl groups, suggesting the participation of flavonoids and other biomolecules in reduction and stabilization processes. The absorption at 1378 cm^−1^ corresponds to C–OH bending, while the peak at 1083 cm^−1^ is assigned to C–O stretching vibrations, both of which highlight the role of polysaccharides and phenolic compounds in nanoparticle stabilization. Importantly, the band observed at 848 cm^−1^ is characteristic of Zn–O stretching vibrations, confirming the successful formation of ZnO NPs. These findings are consistent with previous reports on the green synthesis of ZnO using citrus peel extracts, where hydroxyl, carbonyl, and carboxylate groups were shown to mediate nanoparticle nucleation and growth, while Zn–O related peaks validated the crystalline ZnO phase [41,42,43].

The Rietveld refinement of the XRD pattern of the ZnO sample is presented in Figure 2b. The diffraction pattern of ZnO NPs synthesized from OPE exhibited distinct reflections at 2θ values of 31.8°, 34.4°, 36.3°, 47.5°, 56.6°, 62.8°, 66.4°, 67.9°, 69.1°, 72.5°, and 76.9°. These peaks can be indexed to the (100), (002), (101), (102), (110), (103), (200), (112), (201), (004), and (202) planes of hexagonal wurtzite ZnO, in accordance with the standard JCPDS file No. 36-1451 [16]. These data confirm that the synthesized ZnO crystallizes in a single-phase hexagonal wurtzite structure with the space group P6_3_mc. No secondary phases related to zinc oxide impurities or other oxide phases were detected, indicating the high purity of the prepared sample. The refined lattice parameters, atomic positions, goodness-of-fit value (χ^2^), and reliability factors (R_B_) are summarized in Table 2. These structural parameters are in good agreement with previously reported data for ZnO compounds.

The average crystallite size and the microstrain were evaluated using the Williamson–Hall method according to the following relation:

(1) Bcos(θ)=Kλ/D+4ε sin(θ)

where D represents the average crystallite size, ε is the microstrain of the lattice, B is the full width at half maximum intensity of XRD peaks, k is the Sheerer’s constant (0.90), λ is the X-ray wavelength and θ is the Bragg angle. The plot of Bcos(θ) versus sin(θ) is presented in Figure 2c. From the linear fit of the experimental data, the crystallite size was determined from the intercept, while the microstrain was obtained from the slope. The calculated values indicate an average crystallite size of about 44 nm and a microstrain of 3.4 · 10^−4^. The high intensity of the characteristic peaks in the XRD pattern and the low crystallite size can be attributed to the efficient role of polyphenolic compounds derived from plants as capping and stabilizing agents during biosynthesis. To further confirm particle size in colloidal suspension, DLS analysis was performed, revealing that ZnO NPs in this study exhibit an average particle size of 68.06 nm. This value is smaller than the 75–100 nm range reported by Sarafidou et al. for ZnO NPs synthesized from OP waste [44]. Such a reduced particle size is advantageous for food packaging applications, as smaller NPs generally promote more uniform dispersion within polymer matrices and can potentially enhance barrier and mechanical properties.

Moreover, the biosynthesized ZnO NPs exhibited a negative zeta potential (−21.1 ± 5.42 mV), indicating moderate colloidal stability and favorable dispersion in aqueous systems due to their electrostatic repulsion between particles. This behavior is advantageous for film formulation, as it supports a more homogeneous distribution of NPs within the polymer matrix. In contrast, Radulescu et al. [45] reported a lower absolute zeta potential value (+13.57 mV), suggesting reduced colloidal stability and a greater tendency toward aggregation. This highlights that, although both studies demonstrate the feasibility of synthesizing ZnO NPs from OP waste, the NPs obtained in the present work exhibit improved dispersion behavior, which is critical for achieving consistent performance in food packaging applications.

SEM analysis of the biosynthesized ZnO NPs (Figure 2d) revealed agglomerated structures with distinctive flower-like morphologies. Such hierarchical assemblies are commonly reported in green synthesis routes, where biomolecules from plant extracts guide nucleation and growth, leading to anisotropic architectures. The observed flower-like shapes can be attributed to the self-assembly of ZnO nanocrystals into petal-like structures, driven by the minimization of surface energy and stabilization by residual organic moieties. Agglomeration is typical in biosynthesized ZnO due to strong interparticle interactions and hydrogen bonding mediated by phytochemicals; however, the resulting hierarchical morphology enhances surface area and potential catalytic activity. Similar flower-like ZnO nanostructures have been reported in plant-mediated syntheses, confirming that bioactive compounds act as both reducing and templating agents in directing nanoparticle morphology [18,46].

The UV-Vis absorption spectrum of the biosynthesized ZnO NPs (Figure 2e) displayed a strong absorption band in the UV region, with a distinct edge around 361 nm, followed by a sharp decline in absorbance beyond 400 nm. This behavior is typical of ZnO NPs, indicating their wide band gap and strong excitonic absorption in the near-UV region. The observed absorption edge correlates with a calculated band gap energy of 3.43 eV, aligning with the intrinsic properties of ZnO and confirming the successful formation of the nanoparticles. Additionally, the reflectance profile (Figure 2f) further supports the nanoscale nature of the particles, as the steep edge suggests well-defined electronic transitions characteristic of ZnO nanostructures. Similar absorbance and reflectance behavior has been documented for green-synthesized ZnO NPs, where plant-derived biomolecules modify surface states while maintaining the intrinsic optical properties of ZnO [47,48].

**Figure 3:**
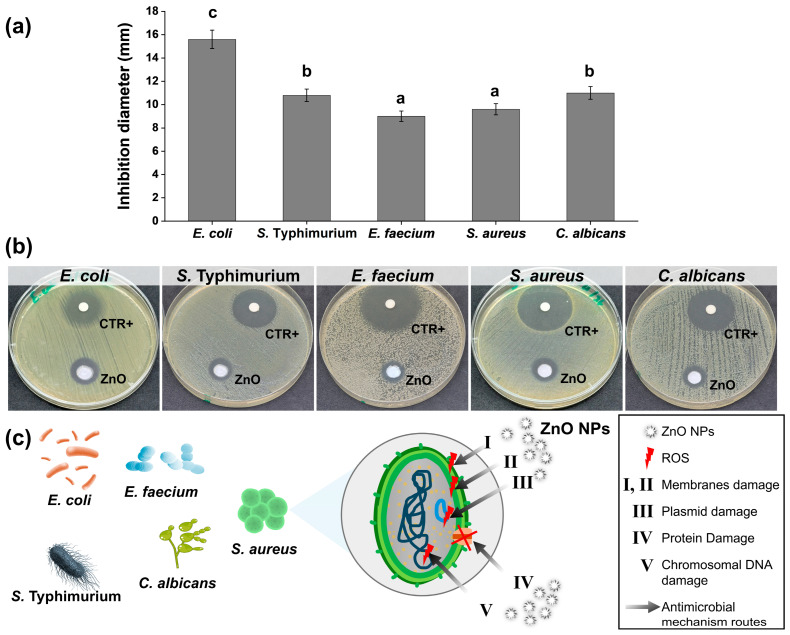
(**a**) Antimicrobial activity of biosynthesized ZnO NPs, expressed as inhibition zone diameters; (**b**) representative images of inhibition zones obtained by the agar diffusion assay for ZnO NPs and the positive control (CTR+, gentamicin); and (**c**) schematic illustration of the proposed inhibition mechanisms. a–c different superscripts indicate significant differences among samples (*p* < 0.05).

ZnO NPs exhibited an antioxidant activity of 40.22 ± 2.20% inhibition in the ABTS assay. This result is consistent with previous studies highlighting the radical quenching capacity of ZnO NPs, which has been attributed to their surface reactivity to stabilize free radicals [49]. The biosynthesized ZnO NPs demonstrated significant antimicrobial activity against both bacterial and fungal strains (Figure 3a). The pronounced inhibition highlights the strong bactericidal effect of ZnO NPs against Gram- and Gram+ strains. The antifungal activity observed against *Candida albicans* further broadens their potential application in food packaging systems. The antimicrobial mechanism of ZnO NPs (Figure 3b) is generally attributed to the generation of reactive oxygen species (ROS), the release of Zn^2+^ ions, and direct interaction with microbial cell membranes (I, II, IV) or intracellular components (III, V) leading to structural damage and cell death. Similar inhibition profiles have been reported in previous studies, where ZnO NPs exhibited broad-spectrum antimicrobial activity against pathogenic bacteria and fungi [50,51].

These characterized OP-derived materials were subsequently used to develop pectocellulosic and nanocomposite films, whose properties are discussed in the following section.

### 2.3. Characterization of the Pectocellulosic and Nanocomposite Films

The properties of the developed films were investigated following a structure–property–function approach, starting from structural characterization (FTIR, SEM), followed by barrier and mechanical properties, and finally functional performance (optical, biological, and biodegradability).

#### 2.3.1. Structural Properties

**Figure 4:**
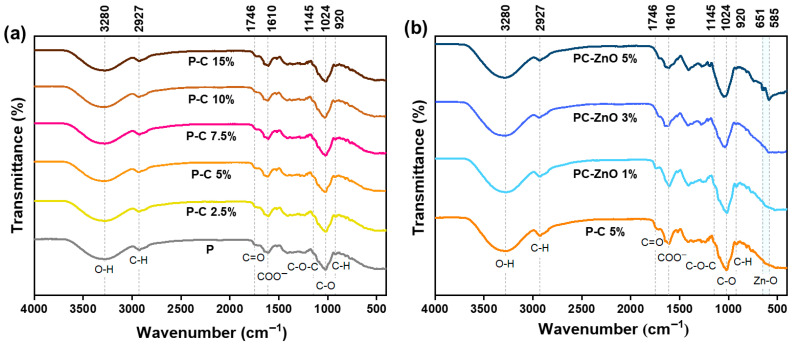
FTIR spectra of (**a**) pectin and pectocellulosic films with varying cellulose content and (**b**) ZnO nanocomposite films based on the optimized pectocellulosic formulation (P-C 5%).

To explore the nature of bonding in the obtained films, FTIR spectroscopy was performed on the pectocellulosic and nanocomposite films, and the absorption spectra in the range of 400–4000 cm^−1^ are shown in Figure 4. The FTIR spectra of the pectocellulosic films, prepared with varying concentrations of OP-derived cellulose were compared to the pectin film. The spectra exhibited no significant alternations in the characteristic absorption bands, with no evident peak shifts or formation of new bands. The distinctive peaks of pectin molecules are present in all pectocellulosic films. The pectin and pectocellulosic films showed two characteristic absorption peaks at 3280 and 2927 cm^−1^ corresponding to -OH stretching vibrations and C-H stretching vibrations, respectively. The two peaks at around 1746 and 1610 cm^−1^ are associated with carbonyl (C=O) stretching of esterified groups and asymmetric stretching of carboxylate ions (COO^−^) in pectin. Furthermore, several bands in the range between 1200 and 950 cm^−1^ correspond to the characteristic bonds of polysaccharides like pectin [52]. No new peaks or significant shifts appear in the FTIR spectrum of pectin–cellulose films compared to pectin film, indicating that the addition of extracted cellulose does not induce detectable covalent chemical modifications in the pectin polymer (Figure 4a). Instead, cellulose acts primarily as a reinforcing filler, contributing to structural enhancement while preserving the chemical integrity of both components. In the same context, Sarafidou et al. [44] developed a pectin-based film reinforced with microfibrillated cellulose (MFC) produced from sugar beet waste for food packaging and found no appearance of peaks in the FTIR spectra between pectin matrix and MFC.

FTIR spectra of ZnO composite films exhibited no noticeable shifts or the appearance of new absorption bands compared to the pectocellulosic film (P-C 5%) (Figure 4b). This observation suggests that the incorporation of nanoparticles at different concentrations into the pectocellulosic film did not lead to significant covalent bond formation within the matrix, which confirms the chemical stability of the pectocellulosic system upon nanofiller incorporation. Consequently, the reinforcement mechanism using nanoparticles is likely determined by interfacial interactions, notably hydrogen bonding and electrostatic attraction, rather than the formation of new chemical bonds in the spectra. Moreover, distinct absorption bands appeared at 651 and 585 cm^−1^, and their intensity increased with ZnO concentration, particularly in the ZnO 5% sample. These peaks are characteristic of Zn–O stretching vibrations, confirming the successful incorporation of ZnO NPs into the polymeric matrix. The increased intensity of these bands at higher ZnO loadings reflects the greater nanoparticle content rather than the formation of new chemical interactions, which is consistent with the absence of significant spectral shifts.

#### 2.3.2. Surface Morphology of Pectocellulosic and Nanocomposite Films

**Figure 5:**
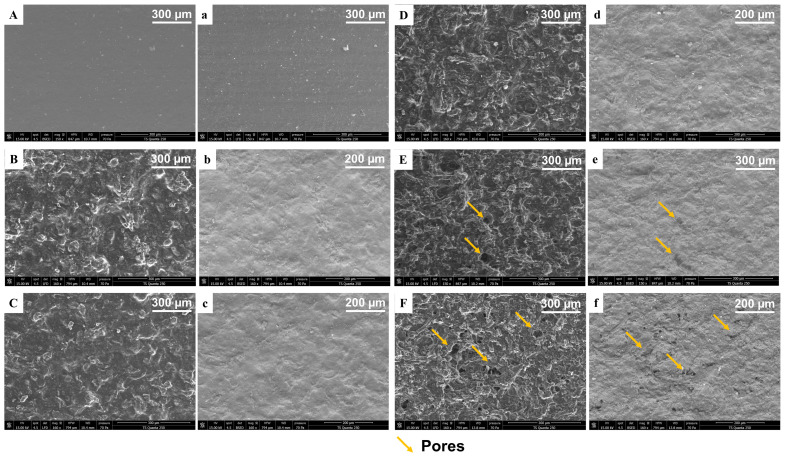
SEM micrographs of pectin (P) and pectocellulosic films containing increasing cellulose contents: P (**A**,**a**), P–C 2.5% (**B**,**b**), P–C 5% (**C**,**c**), P–C 7.5% (**D**,**d**), P–C 10% (**E**,**e**), and P–C 15% (**F**,**f**). Images were acquired using two SEM imaging modes: backscattered electron (BSE) mode to assess overall morphology (**A**–**F**) and secondary electron (SE) mode to examine surface topography in detail (**a**–**f**).

SEM analysis was performed to evaluate the impact of varying cellulose concentration on the film matrix structure. The SEM micrographs (Figure 5) revealed that the pectin control film exhibited a smooth, continuous, and uniform morphology, with no visible defects, confirming the integrity of the pectin matrix (Figure 5(A,a)). Upon cellulose incorporation, the surface became more complex, as cellulose particles could be distinguished within the film structure (Figure 5B–F). Up to 7.5% cellulose, the matrix remained homogeneous and compact (Figure 5D), with smooth surfaces (Figure 5d), indicating good compatibility and dispersion between pectin and cellulose. However, starting at 10% cellulose, a porous structure became evident (marked with yellow arrows), particularly in secondary electron (SE) mode images (Figure 5e), suggesting phase separation and poor filler dispersion. At 15% cellulose, SEM images showed pronounced pores and heterogeneity (Figure 5(F,f)), confirming that excessive cellulose disrupted the continuity of the film matrix. In fact, at higher cellulose concentrations (≥10%), the polymer matrix could not fully accommodate or disperse the excess cellulose. Instead of integrating evenly, cellulose particles aggregated into clusters. These aggregates disrupt the continuity of the film, creating microvoids and pores visible in SEM images. Aggregation reduces interfacial adhesion between pectin and cellulose, leaving gaps that appear as porous structures. This morphological transition highlights the balance between reinforcement and aggregation. Moderate incorporation of cellulose enhances the matrix through physical interactions, matrix densification, and improved filler dispersion. However, at higher loadings, the dispersion threshold is exceeded, resulting in void formation and structural defects. Similar observations have been documented in biopolymer films reinforced with cellulose. Acharya et al. [53] reported that the addition of commercial cellulose to chitosan-based films improved their physical properties at low concentrations (5% *w*/*v*), whereas higher loadings (10–15%) led to less favorable performance. Sharaby et al. [14] similarly observed that commercial pectin-based films reinforced with nanocellulose exhibited more compact morphologies and enhanced mechanical properties at moderate filler content (5%).

**Figure 6:**
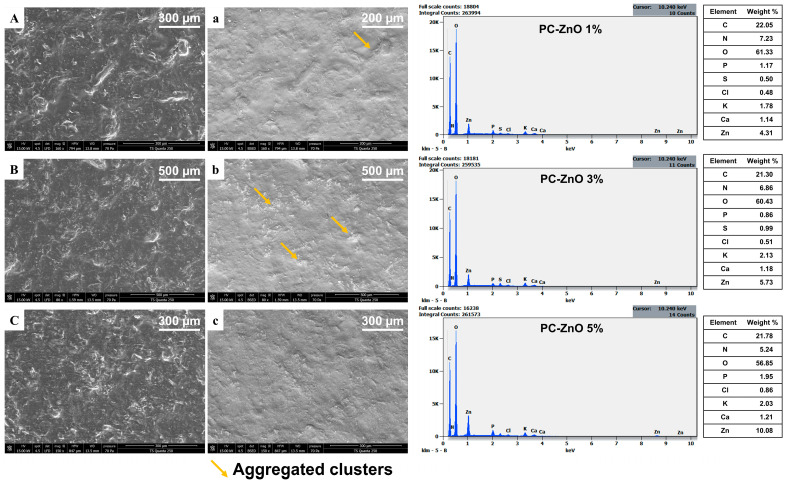
SEM micrographs of pectocellulosic nanocomposite films containing increasing ZnO NP contents: PC-ZnO 1% (**A**,**a**), PC-ZnO 3% (**B**,**b**), and PC-ZnO 5% (**C**,**c**). Images were acquired using two SEM imaging modes: backscattered electron (BSE) mode to assess overall morphology (**A**–**C**) and secondary electron (SE) mode to examine surface topography in detail (**a**–**c**). EDX spectrum of ZnO NP-based films at different concentrations.

Upon the addition of ZnO NPs, the SEM micrographs showed a rough and granular surface (Figure 6). At the lowest ZnO concentration (1%), the NPs tended to form aggregated clusters (marked with a yellow arrow), disrupting the uniformity of the polymer matrix (Figure 6(A,a,B,b)). Interestingly, at higher ZnO loadings (5%), the films exhibited a denser, more compact, and homogeneous morphology (Figure 6(C,c)), closely resembling the optimized cellulose (5%) film. This suggests that at higher concentrations, ZnO became more uniformly dispersed within the pectin–cellulose matrix. This can be attributed to the improved colloidal stability and stronger interactions between ZnO and the biopolymer chains, leading to improved structural integrity [29]. Such behavior has been reported in other biopolymer nanocomposites, where low nanoparticle content promotes localized aggregation due to insufficient stabilization, while moderate concentrations enhance dispersion and matrix compatibility [14].

The EDX spectra of the nanocomposite films (Figure 6) confirmed the elemental composition of the pectin–cellulose matrix, showing dominant carbon and oxygen signals characteristic of polysaccharides. Minor mineral elements originating from the natural biomass were also detected. Importantly, the Zn signal was clearly observed in all ZnO-containing films, and its intensity increased proportionally with ZnO concentration. At 5% ZnO loading, the Zn signal was significantly enhanced, confirming the successful incorporation of NPs within the biopolymer matrix.

These structural observations provide a basis for understanding the evolution of barrier and mechanical properties discussed in the following sections.

#### 2.3.3. Water Barrier Properties

The water barrier properties (Table 3) of the studied films were evaluated in terms of moisture content (MC), water solubility (WS) and water vapor permeability (WVP).

The control pectin film exhibited a MC of 23.41 ± 0.78%, which increased upon cellulose incorporation, reaching 27.52 ± 0.79% at P-C 2.5% and stabilizing around 25–26% between 5 and 15%. The initial increase reflects the hydrophilic nature of cellulose, whose abundant hydroxyl groups provide additional sorption sites for bound water. Similar trends have been reported in pectin–cellulose composites, where cellulose microfibrils introduce polar domains that enhance water uptake and moisture retention [14].

However, the plateau observed between P-C 5% and P-C 15% suggests that as cellulose content increases, the polymer network becomes denser through physical interactions and polymer chain entanglement, thereby reducing free volume and limiting further water absorption. This balance between hydrophilicity and densification has also been described in pectin/cellulose nanocrystal films, where moderate cellulose addition increased bound water but simultaneously restricted mobility, leading to stabilized moisture levels [54].

ZnO NPs further increased MC, reaching 30.87 ± 1.73% in the PC-ZnO 5% film. This increase may be attributed to the surface hydroxyl groups of ZnO NPs, which can interact with water molecules and promote bound water retention within the matrix. Comparable trends have been reported in starch-based nanocomposites containing ZnO, where nanoparticle surfaces contributed to increased moisture affinity [55].

The WS of the control pectin film was 62.06 ± 5.13%, reflecting the hydrophilic nature of pectin (Table 3). Results reported in the literature indicate that pectin films may present variable solubility, e.g., 19% [56] or 52% [57], which can be explained by differences in pectin structure, particularly the degree of methylation. Incorporation of cellulose progressively reduced solubility, reaching minimum values between 5% and 7.5% cellulose (≈54–55%), corresponding to a reduction of approximately 12%. This reduction indicates improved structural integrity and interfacial compatibility between cellulose and pectin, which restricts chain mobility and reduces the fraction of water-extractable components. Comparable results were reported by Tabatabaei et al. [58], who demonstrated that pectin/cellulose nanocrystal films exhibited lower solubility than pristine pectin films, attributing this behavior to improved cohesion and reduced leaching of soluble fractions. At 15% cellulose (P-C 15%), solubility increased sharply to 67.40 ± 0.31%, surpassing the control. This behavior is consistent with SEM observations (Section 2.3.2), which indicate increased porosity and enhanced water penetration. Such behavior has been described in pectin nanocomposites with high filler content, where aggregation disrupts film continuity and increases extractability [11]. These findings highlight a compatibility window between 5% and 7.5% cellulose, beyond which aggregation compromises water resistance. This observation aligns with the structural features discussed in SEM (Section 2.3.2), where excessive filler loading leads to heterogeneous film morphology.

Furthermore, the addition of ZnO NPs exhibited a concentration-dependent pattern (Table 3). At 1% loading, WS was highest (69.45 ± 3.76%), suggesting that low nanoparticle concentrations may disturb polymer–polymer interactions, facilitating film solubilization. WS decreased in films containing 3% and 5%, indicating that higher nanoparticle concentrations enhanced cohesion and reduced water penetration. This trend suggests that sufficient nanoparticle content is required to achieve effective interfacial interaction and matrix stabilization, whereas insufficient loading may induce local heterogeneities. Similar behavior has been reported in biopolymer/ZnO nanocomposites [55,59].

The WVP results confirmed the trend observed in WS (Table 3). In fact, the control pectin film exhibited a WVP of 214.39 ± 20.63 g·mm/m^2^·day·kPa, reflecting the hydrophilic nature of pectin and its relatively open polymer network. Incorporation of cellulose progressively reduced WVP to 187.58 ± 6.10 g·mm/m^2^·day·kPa (P-C 5%) and reached the lowest value of 183.09 ± 7.27 g·mm/m^2^·day·kPa (P-C 2.5%). This steady decline indicates that cellulose reinforcement enhances barrier properties by increasing matrix tortuosity and reducing free volume available for vapor diffusion. The rigid structure of cellulose and its good interfacial compatibility with the pectin matrix promote physical interactions and matrix densification, creating a more compact network that slows water vapor transport. Similar improvements have been reported in pectin–cellulose nanocomposites, where cellulose nanocrystals reduced WVP by strengthening the polymer matrix and lengthening diffusion pathways. At 15% cellulose (P-C 15%), WVP increased to 213.04 ± 7.77 g·mm/m^2^·day·kPa, suggesting that excessive cellulose loading leads to poor dispersion and aggregation, introducing microvoids or weak interfaces that act as preferential channels for vapor transport. Likewise, Tabatabaei et al. [58] reported that while cellulose nanocrystals improved barrier properties at moderate loadings, excessive incorporation compromised film uniformity and led to higher WVP. The results obtained are consistent with SEM observations, which indicated a more uniform and compact matrix at moderate levels of cellulose incorporation (5–7.5%). However, excessive amounts of cellulose led to structural heterogeneity characterized by pore formation and reduced barrier performance. This underscores the importance of controlling filler dispersion and interfacial adhesion to sustain optimal barrier properties.

The incorporation of ZnO NPs led to a progressive decline in WVP values as nanoparticle concentration increased, with PC-ZnO 5% achieving the lowest permeability (181 ± 2.26 g·mm/m^2^·day·kPa). This downward trend indicated that ZnO NPs act as effective nanofillers, reducing free volume and hindering water vapor diffusion through the polymer matrix. The observed reduction in WVP with increasing ZnO concentrations is consistent with previous studies on biopolymer nanocomposites. Abdullah et al. demonstrated that ZnO NPs incorporated into biopolymer films decrease WVP by filling microvoids and enhancing matrix compactness [59].

#### 2.3.4. Mechanical Properties

The incorporation of cellulose into pectin films significantly influenced their mechanical performance (Table 3). The control pectin-based films (P) exhibited relatively low TS (4.03 ± 0.56 MPa) and YM (50.5 ± 6.37 MPa), reflecting their weak and less rigid structure. Upon addition of cellulose, both TS and YM increased significantly (*p* < 0.05), reaching maximum values at 10% cellulose (TS = 10.76 ± 1.25 MPa; YM = 157.3 ± 42.88 MPa). This enhancement can be attributed to the reinforcing effect of cellulose microfibrils, which establish physical entanglement and interfacial interactions with the pectin matrix, thereby improving stress transfer and rigidity. However, at higher cellulose content (15%), both TS and YM decreased significantly (*p* < 0.05) (TS = 4.95 ± 0.43 MPa; YM = 70.1 ± 15.03 MPa), which is consistent with the structural heterogeneity and pore formation observed in SEM analysis (Section 2.3.2), leading to stress concentration points and weakening of the film structure. These structural discontinuities reduce effective stress transfer within the matrix (Figure 5F). Elongation at break (%E) remained relatively stable (~23%) up to 7.5% cellulose but decreased significantly (*p* < 0.05) at higher concentrations (18.9 ± 1.43% at 10% and 16.52 ± 1.43% at 15%), indicating reduced flexibility associated with decreased structural homogeneity at higher filler loadings [60]. Notably, the absence of new covalent bonds in FTIR analysis suggests that the observed mechanical reinforcement arises primarily from physical interactions (e.g., hydrogen bonding and interfacial adhesion) rather than chemical crosslinking, which is consistent with the improved dispersion observed at moderate cellulose content (5–7.5%).

The mechanical properties of pectocellulosic films were further enhanced by the incorporation of ZnO NPs (Table 3). The P-C 5% film (without ZnO) served as the control, exhibiting a tensile strength (TS) of 8.20 ± 1.39 MPa, elongation at break (%E) of 23.7 ± 1.59%, and Young’s modulus (YM) of 131.0 ± 17.15 MPa. Upon the addition of ZnO, a concentration-dependent improvement in mechanical strength and stiffness was observed. At 1% ZnO, TS decreased significantly (*p* < 0.05) to 5.37 ± 0.31 MPa, suggesting that low nanoparticle loading may not provide sufficient reinforcement. However, increasing ZnO to 3% and 5% significantly improved TS (*p* < 0.05) (8.17 ± 0.47 and 10.43 ± 0.60 MPa, respectively) and YM (158.69 ± 5.56 and 163.87 ± 5.74 MPa, respectively), surpassing the control film. These enhancements can be attributed to the homogeneous dispersion of ZnO NPs within the polymer matrix, which promotes strong interfacial interactions with pectin and cellulose chains, thereby improving stress transfer and rigidity. Elongation values remained relatively stable across ZnO concentrations (around 22%), indicating that the films retained flexibility despite increased stiffness. This balance between strength and elasticity is consistent with previous reports on ZnO-reinforced biopolymer films [14,61].

#### 2.3.5. Optical Properties

**Figure 7:**
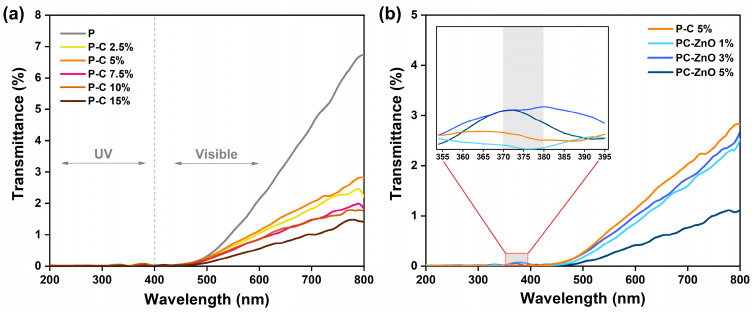
UV-Vis transmittance spectra of (**a**) pectin and pectocellulosic films with increasing cellulose content and (**b**) ZnO-reinforced pectocellulosic films at different nanoparticle loadings. The inset highlights the zoomed part of the ZnO nanocomposite film transmittance in the region of 370–380 nm.

Beyond structural and barrier performance, optical properties are critical for packaging applications, particularly for light-sensitive products. UV–Visible spectra of the pectin matrix, pectocellulosic films, and ZnO composite films were compared, and light transmission in the range of 200–800 nm is presented in Figure 7. All films exhibited very low transmittance across the UV–Visible region, indicating strong light-blocking behavior, likely due to the presence of residual pigments from OP. The pectin film displayed a transmittance of approximately 7%, while the incorporation of cellulose at increasing concentrations further reduced light transmission to ~3% at 2.5% cellulose and ~1.5% at 15% cellulose, indicating exceptional light-blocking capacity. This progressive decrease demonstrates that cellulose incorporation significantly enhances the UV–Visible light barrier properties of the films, particularly across the visible range (400–800 nm) (Figure 7a). The reduction in light transmission can be attributed to increased light scattering caused by cellulose particles dispersed within the matrix, as well as to the higher film opacity resulting from structural densification. Similar trends have been reported in soy protein films incorporating cellulose from licorice residue and gelatin composite films reinforced with cellulose nanocrystals from eucalyptus kraft pulp, which showed enhanced UV-light barrier properties [62,63]. These pectocellulosic films provide effective protection against UV damage while supporting the development of sustainable packaging solutions.

The UV spectra of the optimized pectocellulosic matrix (P-C 5%) (Figure 7b) were significantly influenced by nanoparticle loading (1%, 3%, and 5%). Specifically, the progressive decrease in UV transmittance with increasing ZnO concentration suggests an enhanced UV-shielding capacity of the composite films. This behavior is primarily attributed to the intrinsic UV-absorbing properties of ZnO NPs, which possess a wide band gap (~3.3–3.4 eV) and strong absorption in the near-UV region. A distinct absorption band between 370–380 nm, absent in control P-C 5%, becomes more prominent with increasing ZnO concentration, particularly in PC–ZnO 3% and PC–ZnO 5%, as shown in the inset of Figure 7b. This peak corresponds to the characteristic absorption of ZnO, which generally occurs in the 360–380 nm range [41,43]. Moreover, the presence of ZnO NPs contributes to additional light scattering within the polymer matrix, further reducing transmittance. These results suggest that pectocellulosic films incorporating ZnO NPs have strong potential as active packaging materials for the protection of light-sensitive food products.

The measured opacity values for the pectin-based films demonstrate a clear increasing trend with increasing cellulose concentrations (Table 3). The control pectin film (P) exhibited an opacity of 3.92 ± 0.19. In contrast, the addition of cellulose progressively enhanced opacity, culminating in 5.65 ± 0.19 for the highest concentration tested (P-C 15%). This increase in opacity is consistent with recent literature on polysaccharide-based films, which indicates that the incorporation of cellulose powder or MFC typically results in elevated opacity values. Di Liberto and Dintcheva [64] reported that chitosan composite films enriched with cellulose exhibited significantly greater opacity than chitosan-only controls, due to the light-scattering properties of the cellulose particles. The mechanism underlying the opacity enhancement in pectin–cellulose films is primarily attributed to light scattering by cellulose particles. Cellulosic microstructures, such as microcrystalline cellulose (MCC), MFC or cellulose powder, consist of micro-scale crystalline domains that disrupt the uniformity of the polymer matrix, creating interfaces where incident light is scattered rather than transmitted [65]. This effect is well described by Mie theory, which predicts that particles with dimensions comparable to the wavelength of visible light (400–700 nm) are highly effective scatterers, leading to increased opacity in composite films [66].

The incorporation of ZnO NPs induced significant changes in the opacity of the pectocellulosic films. Compared to the control film (P-C 5%), the incorporation of 1% ZnO resulted in a similar opacity value (4.64), whereas 3% ZnO slightly increased opacity (4.83), and 5% ZnO produced the highest opacity (5.09). This behavior is attributed to the strong light-scattering capacity of ZnO NPs, which reduces transparency by disrupting the uniformity of the polymer matrix [15]. Similar UV-shielding properties of ZnO NPs have been observed in chitosan-ZnO nanocomposites [67], HPMC-ZnO nanocomposites [68], and agar-ZnO and CMC-ZnO nanocomposites [19].

The incorporation of cellulose into OP-derived pectin films resulted in pronounced and systematic changes in colorimetric parameters (Table 3), as evidenced by the progressive increase in L* values from 55.15 ± 4.94 (P, control) to 68.48 ± 2.12 (P-C 15%), alongside decreases in a* (from 22.09 ± 2.50 to 12.52 ± 1.11) and b* (from 75.01 ± 2.17 to 69.4 ± 2.61) values. These results indicate that the films became lighter (higher L), with reduced red (lower a*) and yellow (lower b*) tones, and exhibited lower overall color change intensity at higher cellulose loadings. Such trends are consistent with the established understanding that cellulose addition can significantly alter the optical behavior of biopolymer films through light scattering, chromophore dilution, and microstructural changes [66]. OP-derived pectin may contain residual pigments, such as carotenoids and flavonoids, which impart characteristic red and yellow hues to the films. The addition of cellulose dilutes the concentration of these chromophores per unit volume, leading to decreases in a* and b* values. Furthermore, cellulose fibers can partially mask or encapsulate pigment particles, reducing their optical contribution to the overall film color. This masking effect has been observed in previous studies, where cellulose addition led to more uniform and less intensely colored films, as reported by Moreno et al. [69]. Moreover, the increase in L* values accompanied by reductions in a* and b* can be primarily attributed to enhanced light scattering within the film matrix, a well-documented phenomenon in cellulose-containing systems. Cellulose particles possess dimensions comparable to or larger than the wavelength of visible light, making them efficient scatterers according to Mie theory [66], as also evidenced by the UV-Vis results. As cellulose concentration increases, the density of scattering centers rises, leading to greater diffuse reflectance and reduced color saturation [70]. This effect is further amplified by the heterogeneous morphology of cellulose, which introduces microscale roughness and porosity into the film, as evidenced by SEM results (Figure 5E,F). The relationship between fiber size and optical properties is well established: larger cellulose fibers and aggregates scatter light more effectively, resulting in increased opacity and lightness (L*) but decreased transparency and chromaticity (a*, b*) [65]. The incorporation of ZnO NPs induced significant changes in the color properties of the pectocellulosic films. Compared to the control film (P-C 5%), the addition of ZnO NPs increased L* values to approximately 62 at 1–3% ZnO, indicating a brighter and whiter appearance, while b* values also increased significantly (*p* < 0.05) (78.53 ± 3.31 at 1% and 82.31 ± 1.05 at 5%), reflecting enhanced yellowness. Similarly, the overall color difference (ΔE*ab) increased significantly (*p* < 0.05) with ZnO concentration, reaching 93.88 ± 1.33 at 5%, confirming perceptible visual changes compared to the control [23].

#### 2.3.6. Biological Properties

In addition to physicochemical properties, the functional performance of the films was evaluated in terms of antioxidant and antimicrobial activities.

The antioxidant properties of the films were assessed using ABTS and DPPH radical scavenging assays, revealing distinct trends depending on cellulose and ZnO incorporation (Table 3). Pure pectin films exhibited strong ABTS inhibition (72.73 ± 1.05%) but relatively low DPPH activity (38.71 ± 7.17%), reflecting the well-known difference in sensitivity between the two assays. The addition of cellulose at moderate concentrations (2.5–10%) maintained or slightly enhanced ABTS activity (up to 78.36 ± 5.12% at 10%), while higher cellulose loading (15%) reduced inhibition (66.82 ± 2.88%), which can be associated with the structural heterogeneity observed in SEM analysis (Section 2.3.2), limiting radical accessibility. In contrast, beyond 2.5% cellulose, DPPH inhibition decreased with cellulose addition, dropping to 23–30%, suggesting that cellulose reinforcement may hinder the diffusion of DPPH radicals into the polymer matrix. The observed improvement in ABTS activity at moderate cellulose levels suggests that cellulose reinforcement may enhance the entrapment and stabilization of phenolic compounds within the pectin matrix, thereby promoting radical scavenging. Similar findings were reported by Meerasri et al. [71], who observed that pineapple peel pectin films retained high antioxidant activity due to phenolic entrapment within the biopolymer network. However, the significant decrease of antioxidant activity in cellulose content above 10% is consistent with the reduced structural homogeneity evidenced by SEM (Section 2.3.2), which likely limits the accessibility and release of phenolic compounds. Excess cellulose may also dilute the active pectin fraction, lowering the concentration of bioactive compounds per unit mass of film. Comparable results were reported by Lawal et al. [72] who found that excessive reinforcement in carboxymethylcellulose films reduced antioxidant activity due to phase separation and limited extractability of phenolics. The mechanism underlying these trends is linked to the balance between structural reinforcement and bioactive retention. At moderate cellulose levels, physical interactions and matrix densification create a denser matrix that protects phenolics from degradation and leaching, enhancing radical scavenging. At higher loadings, structural discontinuities (as observed by SEM) reduce phenolic accessibility and limit radical interaction.

The incorporation of ZnO NPs significantly improved ABTS scavenging in a concentration-dependent manner. Films containing 5% ZnO achieved near-complete inhibition (98.20 ± 2.91%), significantly higher than that of cellulose-only control (67.14 ± 0.66%) (*p* < 0.05). This enhancement is consistent with literature reporting that ZnO NPs can act as electron donors and promote radical neutralization, while also catalyzing the generation of antioxidant species through surface redox reactions. However, the effect of ZnO on DPPH activity was less pronounced, with values ranging from 24.37 ± 4.83% (1% ZnO) to 32.26 ± 3.43% (5% ZnO). Although a slight increase was observed at higher ZnO concentrations, DPPH inhibition remained lower than ABTS values, which can be attributed to differences in radical solubility, steric accessibility, and reaction mechanisms between the two assays. These results are consistent with previous studies reporting a dose-dependent enhancement of antioxidant activity in ZnO-based films [20].

The antimicrobial assays revealed selective inhibition depending on both the microorganism and cellulose concentration (Table 3). The control pectin film showed no inhibition against *E. coli*, or *E. faecium*, but exhibited moderate activity against *S.* Typhimurium (7.25 mm). Incorporation of cellulose enhanced antimicrobial performance in specific cases, especially in P-C 5% and P-C 7.5% samples. This increase in activity indicates that cellulose reinforcement improved bioactive retention and microbial interaction. These results suggest that cellulose addition modifies film porosity and phenolic distribution, enhancing the release of active compounds from the pectin matrix. In this context, cellulose acts primarily as a modular structure facilitating phenolic diffusion rather than directly contributing to antimicrobial activity [73]. The mechanism underlying this activity is linked to the phenolic compounds retained in the biopolymers, which are known to disrupt microbial membranes and inhibit growth. At moderate cellulose loadings (5–7.5%), improved dispersion and matrix cohesion likely facilitated controlled release of phenolics, explaining the enhanced inhibition activity. However, beyond 10% cellulose, antimicrobial activity decreased for most strains, which is consistent with the reduced structural homogeneity observed in SEM analysis (Section 2.3.2), limiting the accessibility and release of bioactive compounds. This trend parallels the decrease in TPC and antioxidant activity at high cellulose loadings, confirming that excessive reinforcement compromises functional properties. Comparable findings have been reported in recent studies. Meerasri et al. [71] demonstrated that pectin films from pineapple peel exhibited antimicrobial activity due to phenolic retention, with performance dependent on matrix uniformity. Lawal et al. [72] observed that carboxymethylcellulose films reinforced with date seed components showed enhanced antimicrobial activity at moderate loadings, but reduced efficacy at higher filler content. Similarly, Said & Lee [54] reviewed pectin-based active films and emphasized that antimicrobial performance is strongly correlated with phenolic content and dispersion quality. On the other hand, the incorporation of cellulose and ZnO NPs significantly influenced the inhibition zones against both Gram-positive and Gram-negative bacteria, as well as *C. albicans*. The addition of ZnO NPs significantly amplified antimicrobial efficacy in a concentration-dependent manner. For instance, PC-ZnO 5% films produced inhibition zones of 14.50 ± 2.12 mm against *S.* Typhimurium and *E. coli*, and 15.50 ± 0.71 mm against *E. faecium*, significantly surpassing the cellulose-only control (*p* < 0.05) (P-C 5%). Notably, *S. aureus*, which showed no inhibition with cellulose films, was strongly inhibited by ZnO-based nanocomposite films (up to 16.00 ± 1.41 mm at 5% ZnO), highlighting the broad-spectrum activity imparted by ZnO. These findings are consistent with previous reports demonstrating that ZnO NPs exert antimicrobial effects through multiple mechanisms, including the generation of reactive oxygen species (ROS), disruption of cell membrane integrity, leakage of intracellular contents, and DNA/protein damage [74,75]. The enhanced inhibition observed with increasing ZnO concentration aligns with literature showing dose-dependent antimicrobial activity in pectin–ZnO films designed for food packaging applications [23,24]. Moreover, the synergistic effect between the biopolymeric matrix and ZnO NPs likely facilitates sustained release and close contact with microbial cells, thereby improving efficacy compared to cellulose-only films [76].

#### 2.3.7. Biodegradability

**Figure 8:**
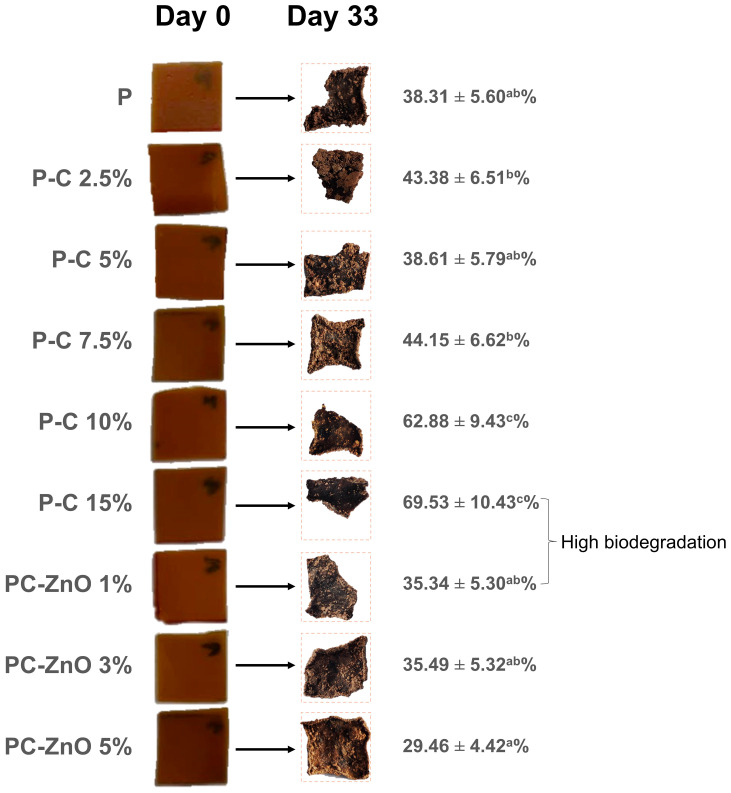
Visual and quantitative assessment of biodegradation of pectocellulosic and ZnO-based nanocomposite films after 33 days under soil burial conditions. a–c different superscripts indicate significant differences among samples (*p* < 0.05).

Finally, the environmental performance of the films was assessed through biodegradability studies (Figure 8). The biodegradability of pectocellulosic films was significantly influenced by both the proportion of OP-derived cellulose and the incorporation of ZnO NPs (Table 3). Pure pectin films (P) demonstrated a moderate biodegradability (38.31 ± 5.60%), reflecting their dense matrix and limited microbial accessibility. The addition of cellulose promoted the films’ biodegradation, with a concentration-dependent effect. At low concentrations (2.5 and 7.5%) the biodegradability slightly increased, whereas the PC-5% film remained comparable to the control. A significant improvement (*p* < 0.05) was observed at higher cellulose concentrations, reaching 62.88 ± 9.43% for P-C 10% and 69.53 ± 10.43% for P-C 15%, indicating that cellulose incorporation significantly promotes microbial degradation. This behavior is consistent with the structural discontinuities and pore formation observed in SEM analysis (Section 2.3.2), which facilitate water diffusion and microbial colonization. Despite the denser fibrillar network formed at higher cellulose concentrations, these structural features enhance accessibility to degrading agents. This overall high degradation (38.31–69.53%) within 33 days is attributable to the hydrophilic nature of pectin and cellulose, which readily absorb water and swell, facilitating microbial colonization and enzymatic hydrolysis. Pectin is particularly susceptible to microbial pectinases, while cellulose is degraded by cellulases, both of which are abundant in soil and compost environments [77]. The combination of these two polysaccharides creates a matrix that is highly accessible to microorganisms, explaining the substantial biodegradation observed. Comparable findings have been reported in recent studies. Zhang et al. [78] demonstrated that pectocellulosic bioplastics derived from fruit waste exhibited rapid biodegradation in soil, confirming the high susceptibility of carbohydrate-based films to microbial attack. The mechanism behind this biodegradation lies in the lack of hydrophobic domains that typically slow microbial access in other biopolymers (e.g., PLA or PBS) [79]. Instead, the predominantly amorphous and hydrophilic structure of pectin–cellulose films allows water penetration and enzymatic hydrolysis. The reinforcing effect of cellulose did not hinder biodegradation; rather, at higher loadings (10–15%), the structural heterogeneity evidenced by SEM enhanced degradation kinetics by increasing matrix accessibility.

Compared to the control P-C 5%, the incorporation of ZnO NPs did not result in a statistically significant reduction in biodegradability. The values obtained for ZnO-containing films (29.46–35.49%) remained statistically comparable to the pure pectin film (38.31 ± 5.60%) and the optimized pectocellulosic formulation. These findings indicate that, despite the known antimicrobial activity of ZnO NPs (Figure 3a), their incorporation did not substantially hinder microbial degradation under the tested conditions. This suggests that the hydrophilic polysaccharide matrix remained accessible to environmental microorganisms and degradative enzymes. A comparable study on cellulose/ZnO nanocomposites reported a similar trend, where ZnO improved functional properties without significantly suppressing biodegradation [6]. While minor variations were observed across formulations, the nanocomposite films still achieved approximately 29–35% degradation within 33 days, confirming their maintained environmental compatibility. Therefore, pectocellulosic-ZnO nanocomposites highlight the delicate balance between structural reinforcement, functional activity, and environmental degradability, supporting their potential as sustainable materials with moderated claims for food packaging applications.

#### 2.3.8. Potential Applications of the Developed Films

From an application perspective, the combined results of mechanical, barrier, optical, antioxidant, and antimicrobial analyses provide valuable insights into the potential use of the developed films in food systems. The moderate water vapor barrier properties and relatively high water solubility suggest that their direct use in high-moisture or liquid foods may be limited without additional structural modifications (e.g., multilayer systems or surface coatings).

However, the films exhibited excellent UV–visible light barrier properties and high opacity, together with significant antioxidant and antimicrobial activities, making them particularly suitable for protecting light-sensitive and perishable food products, such as oils, fresh produce, and processed foods susceptible to photooxidation and microbial spoilage.

Furthermore, the incorporation of ZnO NPs provided enhanced antimicrobial functionality, while the presence of bioactive compounds contributed to antioxidant performance, supporting their application as active packaging materials for short shelf life or dry/semi-moist foods. This suggests that the films are particularly suitable for applications where light protection and microbial control are critical, rather than for high-moisture barrier requirements.

Although the developed films exhibited promising functional properties, further studies on nanoparticle migration and compliance with food safety regulations (e.g., EFSA/FDA guidelines) are required before practical food-contact applications. In particular, ZnO NPs have been reported to partially dissolve under humid or aqueous conditions, releasing Zn^2+^ ions into food simulants. The extent of this migration is influenced by the polymer matrix, nanoparticle size, and environmental factors such as temperature, pH and moisture [80]. Regulatory agencies, including EFSA, have evaluated the safety of ZnO NPs for use in food-contact packaging materials, highlighting the importance of migration testing to ensure consumer safety, and established migration limits for Zn release in food packaging applications (25 mg/kg food) [81].

From an economic and scalability perspective, the proposed approach benefits from the use of low-cost, abundant, and renewable agri-food waste (orange peel) as the primary raw material. The processes involved, including aqueous/ethanolic extraction, alkaline treatment, and green synthesis of ZnO NPs, are relatively simple and potentially scalable. However, further investigations are required to assess the economic feasibility on an industrial scale, including cost analysis, process optimization, life cycle assessment, and comparison with conventional petroleum-based packaging materials.

To further position the proposed materials within the current state of the art, a comparison with recent studies is provided below.

In this study, a single agri-food waste was valorized to provide all key components of the developed packaging film: a pectin-based matrix, extracted cellulose for reinforcement, and green-synthesized ZnO NPs derived from the bioactive extract. This integrated strategy not only advances material innovation but also aligns with circular economy principles by ensuring low cost and an integrated valorization approach with minimized waste generation. Interestingly, the optimal film formulation demonstrated enhanced TS, reduced WVP, and a homogeneous microstructure, indicating potential for practical applications. These improvements highlight the potential of pectocellulosic nanocomposite films as high-performance and sustainable materials. In contrast, other studies have reported improvements in mechanical and antimicrobial properties but relied on commercially sourced pectin, limiting resource integration [14,24]. Likewise, Sarafidou et al. [44] reinforced pectin films with cellulose nanostructures and NPs; however, their ZnO synthesis employed a conventional hydrothermal route in methanol, which is an energy-intensive process less aligned with green chemistry principles. Overall, this work demonstrates that integrated biomass valorization can yield materials with comparable or improved functional performance while enhancing sustainability, thereby advancing the development of next-generation food packaging materials.

## 3. Materials and Methods

The experimental methodology was designed to follow a sequential approach, including raw material preparation, extraction of OP-derived fractions, biosynthesis of ZnO NPs, and subsequent development and characterization of pectocellulosic and nanocomposite films.

### 3.1. Materials

Fresh orange peels (*Citrus sinensis*) were collected as by-products from a local juice processing industry immediately after juice extraction. 2,4,6-tripyridyl-s-triazine (TPTZ), ferric chloride (FeCl_3_·6H_2_O), DPPH (2,2-diphenyl-1-picrylhydrazyl), ABTS [2,2′-azino-bis(3-ethylbenzothiazoline-6-sulfonic acid)] and Trolox (6-hydroxy-2,5,7,8-tetramethylchromane-2-carboxylic acid) were purchased from Sigma-Aldrich (St. Louis, MO, USA). Glycerol, used as plasticizer, and zinc acetate (Zn(CH_3_COO)_2_·2H_2_O) were also purchased from Sigma-Aldrich.

### 3.2. Preparation and Characterization of Orange Peel

The OP samples were first cut into small pieces of approximately 1 × 1 cm to facilitate uniform drying and subsequent grinding. The drying process was carried out in a ventilated oven at 45 °C for 24 h to remove residual moisture while preserving heat-sensitive bioactive compounds. The dried peels were then ground using a Kinematica Polymix PX-MFC 90 D mill (KINEMATICA, Malters, Switzerland), and the resulting OP powder was passed through a 1 mm sieve to obtain a homogeneous particle size distribution. The standardized powder was stored in dark, food-grade containers under dry ambient conditions until further use in extraction and film preparation experiments. This procedure ensured material homogeneity, reproducibility, and limited oxidative or photochemical degradation during storage.

The chemical characterization of the OP lignocellulosic fraction was assessed following standardized procedures to quantify its principal constituents. According to TAPPI T 204 cm-17, the solvent-extractable fraction of OP was determined. In this method, Soxhlet extraction with organic solvents (ethanol/benzene followed by acetone) was performed to isolate low-molecular-weight compounds such as resins, waxes, and tannins. The extractives were recovered by rotary evaporation of the solvent and expressed as a percentage of the oven-dry sample weight [82].

Holocellulose was isolated from extractives-free biomass using sodium chlorite (NaClO_2_) and glacial acetic acid (CH_3_COOH) treatment, following the method described by Álvarez et al. [83]. This procedure, conducted at 96 °C for 90 min, promotes lignin removal while preserving carbohydrate fractions. The cellulose fraction was determined following TAPPI T 212 om-22 by sodium hydroxide treatment of the previously obtained holocellulose, while the hemicellulose content was calculated as the difference between holocellulose and cellulose percentages. This sequential approach allows accurate quantification of holocellulose, cellulose, and hemicellulose fractions in non-woody biomass samples such as OP [84].

Pectin was extracted using a hot acid extraction method, in which the peel powder was treated with dilute acid under heating to solubilize the pectin fraction. The solubilized pectin was then recovered by coagulation with ethanol, following the procedure described by Pokhrel et al. [9]. The precipitated pectin was collected, dried, and quantified gravimetrically as a percentage of the initial dry weight of the sample. Protein extraction was conducted following the method outlined by Deans et al. [85], and the protein content was measured using the Bradford assay [86].

Moisture content was determined by oven-drying at 105 ± 3 °C to constant weight, according to ASTM E1756-08 [87]. Ash content was measured following ASTM E1755-01 [88] by dry oxidation at 575 ± 25 °C in a muffle furnace and was expressed as a percentage of the oven-dry biomass.

The crystalline profile of the OP was examined using an X’Pert PRO diffractometer (PANalytical, Almelo, The Netherlands) equipped with a PW3050/60 goniometer in θ–2θ geometry. Measurements were carried out with Cu Kα radiation (λ = 1.5406 Å) operated at 40 kV and 40 mA. Diffractograms were collected over a 2θ range of 5–60° with a step size of 0.0167° under continuous scanning conditions, using a fixed divergence slit of 0.25°. Data collection and processing were performed with PANalytical Data Collector software.

### 3.3. Preparation and Characterization of OP-Derived Materials and ZnO NPs

**Figure 9:**
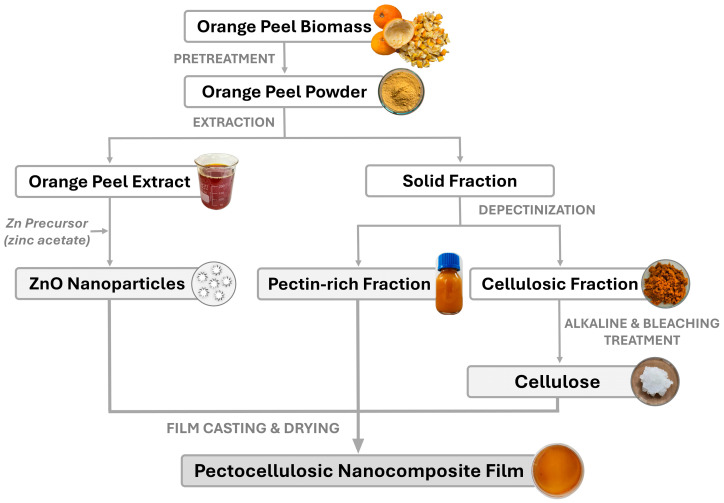
Integrated valorization pathway of orange peel biomass into pectocellulosic-ZnO nanocomposite films.

OP was employed as the sole feedstock for the synthesis of all functional components required for film preparation, ensuring an integrated zero-by-product valorization strategy. From this single biomass source, key fractions were extracted and utilized: the extract was used as a natural reducing agent for the green synthesis of ZnO NPs; the pectin-rich fraction served as the biopolymer matrix, and cellulose acted as a reinforcing agent. This holistic approach maximizes resource efficiency by ensuring that all major fractions of OP contributed to the final material, thereby minimizing waste generation. The workflow of this integrated process is illustrated in Figure 9, and the detailed procedures are described in the following subsections.

#### 3.3.1. Orange Peel Extract (OPE)

OPE was prepared using an aqueous ethanolic solution (60% *v*/*v*) at a ratio of 1:10 (*w*/*v*). The mixture was stirred at 800 rpm for 2 h at room temperature. Subsequently, the solution was ultrasonicated using a probe sonicator for 20 min at 60% amplitude, while being maintained in an ice bath to keep the temperature around 25 °C. The extract was then centrifuged at 6000 rpm for 15 min, followed by vacuum filtration using Whatman No. 1 filter paper (London, UK). Ethanol was removed from the filtrate using a rotary evaporator at 40 °C under reduced pressure. The obtained extract was then lyophilized to obtain a dry powder, which was stored at −20 °C until further use.

The chemical structure of the extract was analyzed using Fourier Transform Infrared (FTIR) spectroscopy with a Bruker Equinox 55 spectrometer (Bruker Co., Ettlingen, Germany), equipped with an attenuated total reflectance (ATR) accessory. Spectra were collected in the range of 4000–400 cm^−1^ with a resolution of 4 cm^−1^, and the sample was scanned 32 times to ensure reproducibility and accuracy.

The TPC of the extract was quantified using the Folin–Ciocalteu assay, following the procedure of Flores-Castañón et al. [89]. Calibration was performed with gallic acid (GA) standards ranging from 10 to 200 µg/mL (R^2^ = 0.998). The reaction was monitored at 725 nm using a FlexA-200 microplate reader (ALLSHENG Instrument Co., Ltd., Hangzhou, China), and results were expressed as milligrams of gallic acid equivalents per gram of dry weight of the extract (mg GAE/g extract).

The antioxidant capacity of the extract was assessed using three complementary assays: DPPH (1,1-diphenyl-2-picrylhydrazyl), ABTS (2,2′-azino-bis(3-ethylbenzothiazoline-6-sulfonic acid)) radical scavenging activity and FRAP (ferric reducing antioxidant power). For this, a solution of OPE was prepared by dissolving 100 mg of extract in 10 mL of a 50% aqueous ethanolic solution. The antioxidant activity via DPPH and ABTS assays was determined following established protocols of Wang et al. [90]. In the present study, these methods were adapted to a microplate format, to allow reduced reagent volumes and high-throughput analysis. For the DPPH assay, a stock solution was prepared in amber glassware by dissolving 2 mg of DPPH in 100 mL of anhydrous ethanol and stirring for 30 min. Subsequently, a dilution from the stock solution was prepared to adjust its absorbance between 0.6 and 1.0 at 517 nm. In the microplate assay, 25 µL of sample solution was added to each well of a 96-well plate, followed by 225 µL of DPPH solution, and the mixture was incubated in the dark for 30 min before measuring the absorbance at 517 nm via 96-well microplate reader (FlexA-200, ALLSHENG, Hangzhou, China). For the ABTS assay, ABTS•^+^ was generated by mixing 7 mM ABTS with 2.45 mM potassium persulfate and incubating the mixture in the dark for 16 h. Afterwards, a dilution was prepared from the stock solution to achieve an absorbance of 0.70 ± 0.02 at 734 nm. In the microplate assay, 25 µL of sample was combined with 225 µL of ABTS•^+^ solution, incubated in the dark for 20 min at room temperature, and the absorbance was recorded at 734 nm. The antioxidant activity via the FRAP assay was determined following the protocol by Khwaldia et al. [91]. The FRAP reagent was freshly prepared by mixing 300 mM acetate buffer (pH 3.6), 10 mM TPTZ in 40 mM HCl, and 20 mM FeCl_3_·6H_2_O in a 10:1:1 ratio. In the microplate assay, 25 µL of sample was incubated with 225 µL of FRAP reagent, at 37 °C for 30 min, and absorbance was measured at 595 nm; reducing power was expressed as µmol Trolox equivalents per gram of extract using a Trolox calibration curve. For all three methods, a Trolox standard curve (25–300 µM) was used for calibration (R^2^ = 0.996 for FRAP, 0.998 for DPPH, and 0.990 for ABTS). Results were expressed as milligrams of Trolox equivalents per gram of extract (mg TE/g extract). Each measurement was performed in triplicate, and results were reported as mean ± standard deviation.

The obtained OPE was subsequently used as a reducing and stabilizing agent for the biosynthesis of ZnO NPs (Section 3.3.3).

#### 3.3.2. OP-Derived Cellulose

OP powder was first subjected to a depectinization step to improve the accessibility of the cellulose fraction. Pectin was removed using an acid-assisted heating method with a 1% acetic acid solution (pH 2.75), and the suspension was heated at 80 °C under magnetic stirring for 2 h. The mixture was then filtered, and the supernatant, rich in pectin, was collected and stored for use as the pectin base (PB) in film preparation. The solid residue, depleted of pectin, was thoroughly washed with distilled water until neutral pH was achieved, thereby preparing it for cellulose extraction. Cellulose extraction was conducted following the method described by Zeng et al. [92]. In brief, the depectinized residue underwent sequential alkaline and bleaching treatments. The alkaline treatment was performed using NaOH (5% *w*/*v*) to remove hemicellulose and other non-cellulosic polysaccharides. This was followed by bleaching with NaClO_2_ (1.4% *w*/*v*) to eliminate lignin and residual pigments.

The structural and morphological characterization of cellulose was conducted using XRD and FTIR, as described in Section 3.2 and Section 3.3.1, respectively. The crystallinity index (CI) was determined from XRD data using the Segal method, according to the following formula:

(2) $$CI(\%)=\frac{\left(I_{200}-I_{am}\right)}{I_{200}}\times 100$$

where I_200_ is the maximum intensity of the (200) peak and I_am_ is the intensity at the minimum between the (200) and (110) peaks, representing the amorphous contribution.

The extracted cellulose and pectin-rich fraction were subsequently used for the formulation of pectocellulosic and nanocomposite films (Section 3.4).

#### 3.3.3. ZnO Nanoparticles

Biosynthesis of ZnO NPs was conducted following the protocol of Chaudhuri and Malodia [93], with minor changes. Briefly, 35 mL of a precursor solution of zinc acetate dihydrate (200 mM) was prepared. Subsequently, 15 mL of OPE (10 mg/mL) was added dropwise to the zinc solution, and the mixture was maintained under continuous stirring for 6 h at room temperature to promote complexation of Zn^2+^ ions with phytochemicals. Then, 50 mL of NaOH solution (1 M) was added gradually under continuous stirring, resulting in the formation of a white Zn(OH)_2_ precipitate. The reaction mixture was incubated overnight at 60 °C, which promoted the thermal conversion of Zn(OH)_2_ into ZnO NPs, assisted by the reducing biomolecules present in the extract. After cooling, the precipitate was centrifuged at 8000 rpm for 10 min and washed repeatedly with distilled water until a neutral pH was reached to remove residual ions. The obtained ZnO NPs were dried at 60 °C to yield a fine white powder.

The characterization of ZnO was performed using FTIR and XRD as described in Section 3.3.1 and Section 3.2, respectively. Samples were observed under high-vacuum conditions at an accelerating voltage of 20 kV. The optical properties of the biosynthesized ZnO NPs were investigated using a PerkinElmer UV–Vis Lambda 365+ spectrophotometer (PerkinElmer, Shelton, WA, USA) equipped for solid-state measurements. Both absorbance and reflectance spectra were recorded in the wavelength range of 200–800 nm. The optical band gap energy (E_g_) was calculated using the direct absorption edge method according to the equation:

(3) $$E_{g}(eV)=\frac{h\times c}{\lambda (nm)}$$

where h is Planck’s constant, c is the speed of light, and λ is the absorption edge wavelength.

Particle size distribution and zeta potential of ZnO NPs were measured using dynamic light scattering (DLS) and electrophoretic light scattering (ELS), respectively, with a Zetasizer Nano ZS (Malvern, UK).

The antioxidant activity of ZnO NPs was quantified using the ABTS assay, as described in Section 3.3. For this, a solution of ZnO NPs (10 mg/mL) was prepared by dispersing ZnO NP powder in DMSO. The data were expressed as a (%) inhibition.

The antimicrobial activity of ZnO NPs was evaluated using the agar diffusion assay against five pathogenic strains: *Escherichia coli ATCC 8739 G(−)*, *Salmonella* Typhimurium *ATCC 14028 G(−)*, *Staphylococcus aureus ATCC 6538 G(+)*, *Enterococcus faecium ATCC 19434 G(+)*, and *Candida albicans ATCC 10231*. A well of 6 mm was aseptically punched into the inoculated agar. The well was then filled with 50 µL of ZnO NPs solution prepared in dimethyl sulfoxide (5 mg/mL). After incubation, the inhibition zones surrounding each well were measured to determine the extent of microbial growth suppression, following the protocol described by Buddhakala et al. [94]. Gentamicin was included as a positive control to validate the assay and provide a benchmark for comparison.

### 3.4. Preparation of Pectocellulosic and Nanocomposite Films

Pectin-based films were prepared using the PB obtained from the depectinization step, with the incorporation of varying concentrations of cellulose (0, 2.5, 5, 7.5, 10, and 15% *w*/*w*) relative to the weight of PB. The formulations were designated as follows: P (control film, PB + 0% cellulose), P-C 2.5% (PB + 2.5% cellulose), P-C 5% (PB + 5% cellulose), P-C 7.5% (PB + 7.5% cellulose), P-C 10% (PB + 10% cellulose), and P-C 15% (PB + 15% cellulose). For each formulation, cellulose was added to the PB solution and stirred at ambient temperature (25 °C) for 1 h to ensure initial dispersion. For the nanocomposite films, the ZnO-based film solutions were prepared by adding ZnO NPs at three different concentrations (1%, 3%, and 5% *w*/*w*, based on the total dry weight of the film) to the previously prepared pectocellulosic formulation (5% *w*/*w*; optimal concentration), with constant stirring for 1 h. The overall formulations were designated as follows: PC-ZnO 1% (PB + 5% cellulose + 1% ZnO), PC-ZnO 3% (PB + 5% cellulose + 3% ZnO), PC-ZnO 5% (PB + 5% cellulose + 5% ZnO). Ultimately, all film formulations were homogenized using an Ultra-Turrax homogenizer for 15 min to achieve uniform distribution of cellulose and nanoparticles within the pectin matrix. Subsequently, glycerol (1% *w*/*w*, based on the total dry weight of the film) was incorporated as a plasticizer and stirred for 30 min.

Film casting was performed by pouring a constant amount of 35 g of each mixture into sterile Petri dishes. The films were dried under controlled ventilation at ambient temperature (25 °C) until complete solvent evaporation.

### 3.5. Characterization of the Pectocellulosic and Nanocomposite Films

#### 3.5.1. Structural and Morphological Analysis

The chemical structure of the pectin-based films and the possible intermolecular interactions between the film components were analyzed using FTIR as described in Section 3.3.

The surface morphology of the films was examined using SEM as outlined in Section 3.4. Micrographs were recorded using two imaging modes. The first mode, backscattered electron (BSE) imaging, was employed to assess structural homogeneity, cellulose and NP dispersion, and overall morphological changes in the pectocellulosic and nanocomposite formulations. The second mode utilized secondary electron (SE) imaging to provide a detailed examination of surface topography induced by increasing cellulose concentrations and the addition of ZnO NPs. This evaluation provided insight into how the incorporation of different concentrations of cellulose and NPs influenced film compactness, porosity, and overall matrix integrity.

#### 3.5.2. Water Barrier Properties

The moisture content (MC) and water solubility (WS) of the pectin-based nanocomposite films were determined according to the procedure described by Baraketi et al. [95]. For MC, films were weighed before and after drying to constant weight, and values were expressed as a percentage of the initial mass. WS was assessed by immersing pre-weighed film samples in 50 mL of distilled water for 12 h at room temperature, followed by drying and re-weighing to calculate the percentage of soluble matter using the equation below:

(4) $$WS(\%)=\frac{W1-W2}{W1}\times 100$$

where W1 is the dried film weight and W2 is the weight of the dried undissolved dried residues.

The water vapor permeability (WVP) of the films was measured gravimetrically in triplicate following the ISO 2528 standard [96]. Film specimens were mounted on permeation cells containing silica gel and placed in a controlled humidity chamber maintained at 38 °C and 90% relative humidity. The water vapor transmission rate (WVTR) was obtained from the slope of the weight gain curve (g/day), normalized by the exposed film area (m^2^). WVP was then calculated using the following equation:

(5) $$WVP(g\cdot mm\cdot m^{-2}\cdot {day}^{-1}\cdot {kPa}^{-1})=\frac{WVTR\times T}{∆P}\times 100$$

where T is the film thickness (mm), and ΔP is the water vapor partial pressure difference across the film (kPa).

#### 3.5.3. Mechanical Properties

Film thickness was measured using a digital micrometer. For each film, ten random measurements were recorded, and the average thickness was calculated. The mechanical properties of the films, including tensile strength (TS), elongation at break (%E), and Young’s modulus (YM), were determined using a universal testing machine (Lloyd, AMETEK STC, Bagnor Regis, UK) under controlled conditions (23 ± 1 °C, 50 ± 5% RH) according to the international standard ISO 291 (2008) [97]. Sample films were cut into 15 mm × 100 mm strips and analyzed in accordance with both ISO 527-1 (2019) [98] and ISO 527-3 (2018) [99]. The mechanical properties represent the mean of five measurements per film.

#### 3.5.4. Optical Barrier Properties

The optical transmittance of the pectocellulosic films was measured in the UV–visible range (200–800 nm) using a UV–Vis spectrophotometer (Optizen 2120 UV, Neogen, Sejong, Republic of Korea). Spectral data were recorded to evaluate the transparency of the films and the influence of cellulose and ZnO NP incorporation on their light-barrier properties.

Film opacity was calculated from the absorbance values at 600 nm, normalized by film thickness. This parameter provided a quantitative measure of the films’ ability to block visible light, which is relevant for packaging applications requiring protection against photo-oxidative degradation.

The color attributes of the pectocellulosic nanocomposite films were evaluated using a Konica Minolta Chroma Meter CR-400 (Konica Minolta Chroma Co., Osaka, Japan). The parameters recorded included L* (lightness), a* (red–green coordinate), and b* (yellow–blue coordinate), which together describe the visual appearance of the films in the CIELAB color space. For each formulation, ten measurements were performed at different points across the film surface to ensure representative values and minimize variability. The overall color difference (ΔE) was calculated using the software relative to the white baseline.

#### 3.5.5. Biological Properties

The antioxidant activity of the pectocellulosic nanocomposite films was quantified using ABTS and DPPH assays, following the procedure described in Section 3.3.1. For this, a weighed portion of film was extracted by maceration in 50% (*v*/*v*) ethanol for 24 h. The extracts were centrifuged and stored in amber glassware for further analysis. For the DPPH assay, 25 μL of the previously prepared film solution was incubated with 225 μL of DPPH reagent, and the absorbance at 517 nm was recorded as A_sample_. On the other hand, the absorbance of 25 μL of a 50% ethanol solution mixed with 225 μL of DPPH was measured and recorded as A_control_. The absorbance of 25 μL of the film solution incubated with 225 μL ethanol was measured and recorded as A_correction_. The results were expressed as percentage inhibition (%), calculated according to Equation (6):

(6) $$DPPH\left(\%\;inhibition\right)=\left(1-\frac{A_{sample}-A_{correction}}{A_{control}}\right)\times 100$$

The same procedure was conducted for ABTS assays by measuring the corresponding absorbances at 734 nm. The results were expressed as percentage inhibition (%), calculated according to Equation (7):

(7) $$ABTS\left(\%\;inhibition\right)=\left(1-\frac{B_{sample}-B_{correction}}{B_{control}}\right)\times 100$$

where B_sample_ is the absorbance of the film solution mixed with the ABTS reagent, B_correction_ is the absorbance of the film solution incubated with ethanol to replace the absent volume of ABTS reagent, and B_control_ is the absorbance of the mixture of ABTS reagent with 50% ethanol without the film solution.

The antimicrobial activity of the pectocellulosic films was evaluated using the agar diffusion assay. Films were cut into circles of 6 mm and placed directly onto the inoculated agar to test them against the five microbial strains as described in Section 3.4.

#### 3.5.6. Biodegradability

The biodegradability of the pectocellulosic films was assessed under soil burial conditions. Film specimens (10 × 10 mm) were placed at a depth of 5 cm in aerated, non-sterile soil collected from the Sidi Thabet perimeter [100]. To ensure microbial activity, soil moisture was maintained by periodically adding distilled water. Samples were incubated at ambient temperature under aerobic conditions, simulating natural environmental exposure. After incubation, films were recovered every 48 h, gently cleaned to remove soil particles, dried at 70 °C for 2 h, and weighed. The extent of biodegradation was expressed as the percentage of mass loss relative to the initial weight, and all experiments were conducted in triplicate to ensure reproducibility.

### 3.6. Statistical Analysis

All experimental procedures were carried out in triplicate, and the results are presented as mean values ± standard deviation (SD). Statistical evaluation was performed using SPSS software, version 22.0 (IBM Corp., Armonk, NY, USA). Differences among treatments were considered statistically significant at a confidence level of *p* ≤ 0.05. Duncan’s multiple range test was applied to compare group means and identify specific differences between film formulations.

## 4. Conclusions

This work demonstrated the feasibility of producing pectocellulosic nanocomposite films from OP biomass through an integrated valorization strategy, yielding multifunctional materials reinforced with cellulose and biosynthesized ZnO NPs. The pectin-rich fraction was used as the film-forming matrix, while extracted cellulose acted as a reinforcing agent, enabling efficient utilization of the biomass.

Moderate cellulose incorporation (5%) provided the optimal balance of properties, increasing TS by approximately 103% and reducing WVP by about 12.5%, while maintaining structural homogeneity. In contrast, higher cellulose loadings (≥10%) induced pore formation and led to reduced performance.

The incorporation of ZnO NPs further enhanced functional performance in a concentration-dependent manner. At 5% loading, nanocomposite films exhibited the highest stiffness (YM = 163.87 ± 5.74 MPa), near-complete antioxidant activity (98.20 ± 2.91% ABTS inhibition), and strong antimicrobial activity (inhibition zones up to 16.00 ± 1.41 mm), while maintaining biodegradability (29.46 ± 4.42% after 33 days).

Overall, these results confirm that OP-derived materials can be transformed into high-performance, biodegradable films with active functionality. However, further investigations are required to assess their suitability for food-contact applications, particularly regarding nanoparticle migration and regulatory compliance.

## Figures and Tables

**Table 2:** Structural parameters obtained from Rietveld refinement of the XRD pattern of ZnO nanoparticles.

Atom	Wyckoff Site	x	y	z
Zn	2b	0.3333	0.6667	0.0024
O	2b	0.3333	0.6667	0.3814

a = 3.2510 (1) Å, b = 3.2510 (1) Å, c = 5.2089 (2) Å, X^2^ = 2.12; R_B_ = 2.62.

**Table 3:** Physicochemical, mechanical, optical, antimicrobial, antioxidant and biodegradability properties of pectocellulosic and ZnO nanocomposite films.

Films	P	P-C 2.5%	P-C 5%	P-C 7.5%	P-C 10%	P-C 15%	PC-ZnO 1%	PC-ZnO 3%	PC-ZnO 5%
Thickness (µm)	436.67 ± 4.16 ^de^	431.00 ± 9.90 ^d^	407.33 ± 5.03 ^b^	440.00 ± 1.00 ^e^	417.33 ± 2.08 ^c^	411.33 ± 2.31 ^bc^	381.90 ± 15.67 ^a^	372.86 ± 0.00 ^a^	381.90 ± 15.67 ^a^
Water barrier properties									
MC (%)	23.41 ± 0.78 ^a^	27.52 ± 0.79 ^c^	26.56 ± 0.32 ^bc^	25.60 ± 0.37 ^b^	25.42 ± 0.11 ^b^	25.77 ± 0.92 ^b^	28.81 ± 0.99 ^de^	29.10 ± 0.44 ^e^	30.87 ± 1.73 ^f^
WS (%)	62.06 ± 5.13 ^b^	58.62 ± 1.87 ^ab^	55.52 ± 2.06 ^a^	54.66 ± 2.28 ^a^	55.79 ± 4.48 ^a^	67.40 ± 0.31 ^b.c^	69.45 ± 3.76 ^d^	59.39 ± 3.57 ^ab^	54.79 ± 11.72 ^a^
WVP (g·mm/m^2^·day·kPa)	214.39 ± 20.63 ^ab^	183.09 ± 7.27 ^a^	187.58 ± 6.10 ^a^	190.86 ± 8.44 ^ab^	201.14 ± 11.11 ^ab^	213.04 ± 7.77 ^b^	186.26 ± 4.93 ^ab^	184.28 ± 8.96 ^a^	181.18 ± 2.26 ^a^
Mechanical properties									
TS (MPa)	4.03 ± 0.56 ^a^	7.23 ± 0.62 ^c^	8.20 ± 1.39 ^c^	7.82 ± 1.03 ^c^	10.76 ± 1.25 ^d^	4.95 ± 0.43 ^ab^	5.37 ± 0.31 ^b^	8.17 ± 0.47 ^c^	10.43 ± 0.60 ^d^
%E	22.98 ± 1.85 ^b^	23.22 ± 2.68 ^b^	23.73 ± 1.59 ^b^	23.25 ± 1.57 ^b^	18.90 ± 1.43 ^a^	16.52 ± 1.43 ^a^	22.41 ± 1.46 ^b^	21.64 ± 1.32 ^b^	22.16 ± 1.70 ^b^
YM (MPa)	50.5 ± 6.37 ^a^	107.4 ± 8.65 ^b^	131.0 ± 17.15 ^b^	123.5 ± 25.90 ^b^	157.3 ± 42.88 ^c^	70.14 ± 15.03 ^a^	123.81 ± 4.34 ^b^	158.69 ± 5.56 ^c^	163.87 ± 5.74 ^c^
Optical properties									
L*	55.15 ± 4.94 ^a^	58.57 ± 4.21 ^b^	58.16 ± 2.42 ^ab^	61.07 ± 2.11 ^b^	61.36 ± 3.17 ^b^	68.48 ± 2.12 ^c^	62.37 ± 2.61 ^c^	62.77 ± 2.13 ^c^	61.03 ± 1.97 ^bc^
a*	22.09 ± 2.50 ^d^	20.17 ± 2.45 ^cd^	20.86 ± 1.34 ^c^	17.49 ± 1.88 ^b^	17.27 ± 2.44 ^b^	12.52 ± 1.11 ^a^	21.56 ± 2.03 ^de^	18.82 ± 1.52 ^bc^	20.15 ± 1.56 ^cd^
b*	75.01 ± 2.17 ^c^	73.66 ± 1.31 ^bc^	74.51 ± 0.94 ^c^	72.15 ± 2.08 ^b^	68.23 ± 4.25 ^a^	69.40 ± 2.61 ^a^	78.53 ± 3.31 ^d^	80.69 ± 2.06 ^e^	82.31 ± 1.05 ^e^
dE*ab	90.29 ± 2.83 ^c^	88.05 ± 3.07 ^c^	88.31 ± 1.93 ^c^	83.23 ± 3.08 ^b^	77.63 ± 8.31 ^a^	77.28 ± 2.46 ^a^	89.40 ± 4.43 ^c^	90.13 ± 2.49 ^c^	93.88 ± 1.33 ^d^
Opacity	3.92 ± 0.19 ^a^	4.75 ± 0.09 ^b^	4.78 ± 0.15 ^b^	4.74 ± 0.30 ^b^	4.88 ± 0.04 ^b^	5.65 ± 0.19 ^c^	4.64 ± 0.02 ^b^	4.83 ± 0.06 ^b^	5.09 ± 0.71 ^b^
Antimicrobial properties (mm)									
*C. albicans*	7.00 ± 0.00 ^a^	7.00 ± 0.35 ^a^	8.25 ± 0.35 ^b^	7.50 ± 0.71 ^ab^	7.50 ± 0.71 ^ab^	No inhibition	6.50 ± 0.00 ^a^	10.25 ± 0.35 ^c^	12.50 ± 0.71 ^d^
*E. coli*	No inhibition			7.00 ± 0.35 ^a^	8.25 ± 0.35 ^b^	7.00 ± 0.35 ^a^	6.75 ± 0.35 ^a^	10.75 ± 1.06 ^c^	14.50 ± 0.71 ^d^
*S.* Typhimurium	7.25 ± 0.35 ^a^	7.50 ± 0.00 ^a^	7.75 ± 1.06 ^a^	8.75 ± 0.35 ^ab^	8.00 ± 0.71 ^a^	7.25 ± 0.35 ^a^	6.75 ± 0.35 ^a^	10.75 ± 1.06 ^b^	14.50 ± 2.12 ^c^
*E. faecium*	7.50 ± 0.00 ^a^	10.50 ± 2.12 ^ab^	11.00 ± 1.41 ^ab^	13.00 ± 4.24 ^bc^	8.50 ± 2.12 ^a^	8.00 ± 0.41 ^a^	7.25 ± 0.35 ^a^	13.25 ± 1.06 ^bc^	15.50 ± 0.71 ^c^
*S. aureus*	No inhibition				9.50 ± 2.12 ^a^	12.00 ± 1.41 ^b^	9.50 ± 2.12 ^a^	12.00 ± 1.41 ^b^	16.00 ± 1.41 ^c^
Antioxidant properties (% inhibition)									
ABTS	72.73 ± 1.05 ^ab^	75.20 ± 4.48 ^b^	67.14 ± 0.66 ^a^	67.78 ± 3.24 ^a^	78.36 ± 5.12 ^b^	66.82 ± 2.88 ^a^	77.54 ± 2.07 ^b^	87.99 ± 3.29 ^c^	98.20 ± 2.91 ^d^
DPPH	38.71 ± 7.17 ^b^	50.54 ± 5.19 ^c^	26.42 ± 4.78 ^a^	30.57 ± 8.26 ^ab^	23.20 ± 1.30 ^a^	23.50 ± 1.09 ^a^	24.37 ± 4.83 ^a^	27.50 ± 1.52 ^a^	32.26 ± 3.43 ^ab^

The data are shown as mean values ± s.d. Data were compared using Duncan’s multiple range test for post hoc comparison (*p* < 0.05). a–f different superscripts within a row indicate significant differences among samples (*p* < 0.05).

## Data Availability

The original contributions presented in this study are included in the article. Further inquiries can be directed to the corresponding author.

## Abbreviations

The following abbreviations are used in this manuscript:

OP	Orange peel
OPE	Orange peel extract
ZnO NPs	Zinc oxide nanoparticles
